# Fertility resilience varies by socioeconomic status and sex: Historical trends in childlessness across 150 years

**DOI:** 10.1016/j.isci.2024.110227

**Published:** 2024-06-09

**Authors:** Milla Salonen, Mirkka Lahdenperä, Anna Rotkirch, Virpi Lummaa

**Affiliations:** 1 Department of Biology, University of Turku, Turku, Finland; 2 Population Research Institute, Väestöliitto, The Family Federation of Finland, Helsinki, Finland

**Keywords:** Socioeconomic status, Natural sciences, Biological sciences, Social sciences

## Abstract

Fertility dynamics are key drivers of demographic change in a population. Fertility resilience is likely to vary by socioeconomic class, yet little investigated. Using a unique dataset tracking the reproduction of family lineages for 150 years, we explored childlessness by socioeconomic status and sex during the demographic transition and recurring societal and economic disturbances in Finland. Lifetime childlessness doubled from the 1800 birth cohort to the 1945–1949 cohort. Higher socioeconomic status (SES) indicated higher lifetime likelihood to reproduce. The fluctuations in childlessness over time appeared to be driven by the low socioeconomic group, showing low fertility resilience. In contrast, a steady increase was seen in high and moderate SES. Our findings suggest that the family formation of lower socioeconomic groups suffers the most during crises and does not necessarily recuperate. Preventing inequalities in family formation and reproduction should be recognized as a key challenge for population resilience to crises.

## Highlights

• Lifetime childlessness doubled from 1800–1804 birth cohort to 1945–1949 cohort
• Fluctuations in childlessness were driven by the low socioeconomic group
• High and moderate socioeconomic groups showed a stable increase in childlessness
• Results indicate low fertility resilience in the low socioeconomic group

## Graphical abstract

**Figure:**
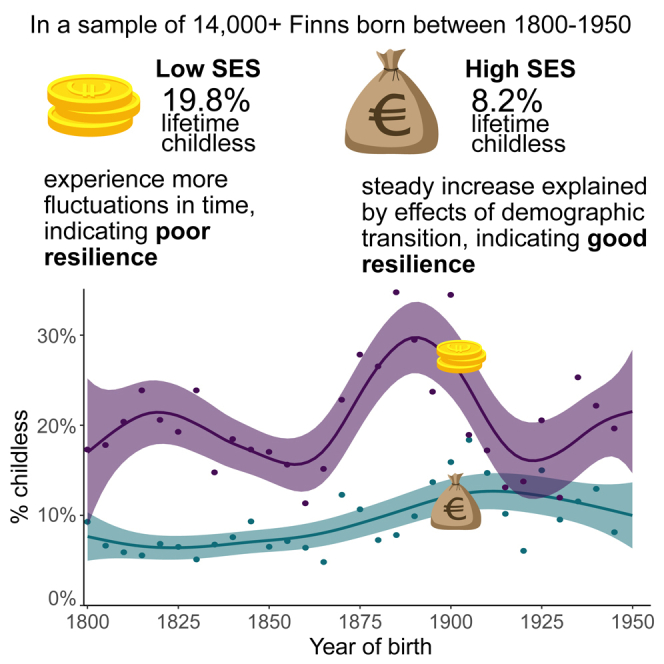

## Introduction

Large societal crises such as famine, war, and disease tend to disrupt population dynamics. The ability of populations or subpopulations to resist, adapt to, and recover from crises that alter the demographic structure can be called demographic resilience.^1^ After a disturbance, resilient populations react less and are quicker to revert to the original stable state. An intense disturbance can also push a population over a tipping point, resulting in the population adopting a new stable state instead of reverting to the original level.^1^ In humans, demographic resilience additionally encompasses institutional settings and policy measures which may both instigate or prevent and mitigate crises.^2^

One important part of demographic resilience is fertility resilience, or stability of fertility dynamics during disturbances. Fertility resilience has only recently emerged as a concept in the human sciences.^3^ The most extreme end of disrupted fertility dynamics is childlessness. Yet, although childbearing and especially failure to reproduce are key drivers of demographic change, we lack studies of how resilient the fertility of different subgroups in a population is in response to crises over larger time spans. We define here fertility resilience as stability of birth rates during times of crises, or a quick recovery to pre-crises fertility trends. A key dimension of fertility resilience is the likelihood to reproduce at all, measured as lifetime childlessness. Understanding individual differences stemming, for example, from socioeconomic disadvantage, is vital to grasp the drivers of such fertility resilience. However, existing research on socioeconomic gradients in reproduction has tended to focus either on specific crises or on the long haul of the demographic transition, rarely combining both.

Fertility dynamics are affected by both long-term trends and societal disturbances. Birth rates have steadily decreased in most countries during the gradual spread of the demographic transition, or the shift from high mortality and fertility to low mortality and fertility^4^ that started in Western Europe at the beginning of the 19th century.^5^^,^^6^ Based on an increase in association between fertility rate and childlessness, childlessness is becoming an increasingly important component of low fertility.^7^ Western European countries and the United States have exhibited similar historical trends in childlessness.^8^^,^^9^^,^^10^ In these countries, the proportion of lifetime-childless women in the early 19th century birth cohorts was 15–20 percent, followed by a subsequent increase to 20–30 percent in childlessness until the 1905–1910 birth cohorts; male childlessness has been much less studied (but see Rotkirch at al.^11^ for trends between 1930 and 1979). After this, childlessness decreased and was at its lowest, 5–15 percent in the 1930–1940 birth cohorts before rapidly increasing again in many Western countries and reaching 12–23 percent in the 1960 birth cohort with the increase expected to continue.^8^^,^^9^^,^^10^ Many reasons have been implicated for fertility decline and increasing proportions of childlessness, including, for example, the demographic transition, wars, economic hardships, urbanization, changes in the prevalence of marriage and age of marrying, and social acceptance of birth control and childlessness.^9^^,^^10^^,^^12^^,^^13^ For example, after the Second World War, earlier marriage underlay the temporal decrease in childlessness.^12^^,^^13^ Thus, various obstructions to entering a union, which in Europe historically often occurred around peak fecundity or age 25, can strongly influence failure to reproduce.^9^^,^^10^ Within marriage, possible reasons for remaining childless include, for example, biological infertility, postponing childbearing to less-fertile later ages, and birth control.^9^^,^^10^ The demographic transition and societal modernization brought a shift from controlling age at first birth by marriage to controlling it within marriage via birth control.

In addition to the centuries-long demographic transition, abrupt social crises may influence childlessness and test fertility resilience. Notably, the First World War and the Great Depression were implicated in the increase in lifetime childlessness in the 1890–1900 birth cohorts in many Western European countries and the United States, through death of potential and current husbands in the army and postponement of childbearing due to economic hardships.^8^^,^^9^ The aftermath of the Second World War, by contrast, witnessed an uptick of fertility rates and lower proportions of childlessness during the baby booms in many countries experiencing rapid economic growth.^12^ In more recent times, the onset of the COVID-19 pandemic caused a quick drop in birth rates in the most affected countries, but a rapid increase in others, followed by a return to pre-crises trends in most cases.^3^^,^^14^ It is important to note that both a sudden growth and decrease in birth rates can challenge demographic resilience and sustainable population dynamics.^15^ Currently, as global fertility continues to decline at an unexpected pace,^16^ while different societal crises caused by both technological change and climate crises are likely to multiply and escalate, the question of fertility resilience is increasingly topical.

Both the long-term demographic transition and abrupt social crises are likely to affect different subgroups of a population differently. Famines and wars are known to cause higher mortality among males and among the poor.^17^^,^^18^ Trends in childlessness can also vary by both sex and socioeconomic status (SES). While children have historically been born predominantly in wedlock, trends in childlessness can differ between men and women through differences in mortality as well as in the likelihood to marry and remarry. In most known societies, men have higher reproductive variance and higher proportions of childlessness compared to women.^19^^,^^20^ SES can influence childlessness in opposite ways: on the one hand, individuals with low SES may have poorer health and lower chance to marry, but, on the other hand, individuals with high SES may have had better access to fertility control.^9^^,^^10^^,^^21^^,^^22^ Before the demographic transition, wealthier families commonly had more children than poor families.^23^ As the shift to longer life expectancy and lower fertility unfolded, the relationship often reversed, so that especially women of lower SES had more children compared to women with higher SES during the 20^th^ century.^24^ Currently, as the demographic transition draws to a close, the socioeconomic gradient appears to be turning positive once again in many countries. For instance, in some Nordic countries, high education is currently associated with higher cohort fertility among both sexes, and also elsewhere childlessness is increasing among men and women with low education.^25^ Fertility decline globally is related especially to postponement of parenting to later ages and increases in lifetime childlessness. This stresses the need for longitudinal studies examining the differences in fertility resilience between SES groups in response to crises and hardships.

In this study, we investigate how fertility resilience differs between SES groups and by sex. We explore fertility resilience by examining the historical trends and variation in childlessness in Finland, a country that has one of the world’s longest continuous registry data spanning from the early 18th century onwards. These registers were maintained by the Lutheran Church and include the births, deaths, occupations, and marriages, allowing us to construct the whole life course of individuals and spanning several family generations. Our study period covers the lifetime reproduction of birth cohorts between 1800 and 1950. At the beginning of this period, Finland was part of Sweden, but annexed by Russia in 1809 after the Finnish War 1808–1809. The Finnish War increased mortality among young men and may have influenced childbearing of young adults born before our study period. In 1832–1833, crop failures and epidemics of dysentery and typhoid increased mortality in Finland.^26^ This crisis may have influenced the fertility of both those who were young children at the time and young adults, born in 1805–1815. In 1866 to 1868, Finland experienced the worst famine since the 17^th^ century. An estimated 150,000 people died due to starvation and infectious diseases. Mortality was high especially among the poor. Again, this crisis may potentially influence the fertility in those who were young children or born during the famine, and of young adults born in 1840–1850.

Finland remained as an autonomous Grand Duchy of Tsarist Russia until its declaration of independence in 1917. Finland did not take part in the World War I (WWI), but in 1918 the Finnish Civil War took place, resulting in excess deaths in men. The Civil War could influence the childbearing of especially young women born at around 1890–1900. The Civil War was followed by the Spanish flu epidemic between 1918 and 1920, which resulted in excess deaths especially in the adult population and could influence fertility in both young adults (born at around 1895–1905) and children (born 1915–1920). World War II (WWII) would likely influence childbearing of young adults (born at and after 1925).

Industrialization and the demographic transition began in Finland comparatively late for Europe, in the 1870s.^27^^,^^28^^,^^29^ During nearly the whole study period Finland was mostly an agrarian society: as late as in the 1950s, nearly half of the population still received their income from agriculture.^30^ Overall, the study period and population offer outstanding opportunities to address how repeated social crises of different types affect fertility resilience over a long period of time. Based on earlier studies we expect the lower SES groups to show more variation in childlessness over time, indicating poor resilience. Based on studies that have revealed higher reproductive variance and childlessness in men,^19^^,^^20^ we also expect childlessness to be more common in men than women. Finally, we expect that trends in childlessness are linked to trends in marriage and family size, as childlessness may result from difficulty finding a spouse or fertility control within marriage.^8^^,^^9^^,^^10^^,^^12^^,^^13^

## Results

**Figure 1:**
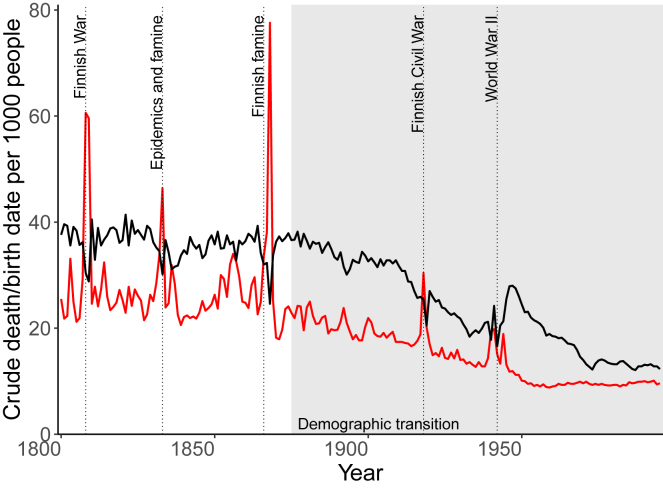
Birth and death rates in Finland, 1800–1995 Crude birth rate in black and crude death rate in red per 1,000 people. Demographic transition in gray area. Data from Statistics Finland^34^^,^^61^ and information of crises from Turpeinen 1975.^26^

We studied lifetime childlessness of individuals born between 1800 and 1950. During these 150 years, Finland underwent the demographic transition from high mortality and fertility to low mortality and fertility as revealed from the crude birth and death rates and encountered several societal crises (Figure 1).^26^ A Similar change was reflected in the total fertility rate and life expectancy at birth: until 1900, the total fertility rate stayed at around 4.8 children per woman, after which it started to decrease and dropped permanently below two in 1970.^31^ At the start of the study period, life expectancy at birth was 30.4 years for men and 33.2 years for women.^32^ In contrast, at the end of the study period in 1950, life expectancy at birth had nearly doubled, being 58.4 years for men and 65.8 years for women.^32^

### Historical trends in lifetime childlessness

We assessed lifetime childlessness among individuals that survived until 45 years of age and whose entire life history was known, meaning that we had records of all their children. Individuals that did not have a record of any children were considered childless. Some individuals had missing information in, for example, SES, and, thus, the sample size varied between 14,408 and 17,569 (Table S1). We fitted generalized additive models (GAMs) to evaluate the significance of temporal trends. A large chi-squared statistic and a small *p* value indicate that childlessness (or underlying factors) increased, decreased, or fluctuated in time. The effective degrees of freedom (df) (edf) reflect the degree of non-linearity, with edf >2 indicating strong non-linearity in the trend.

**Figure 2:**
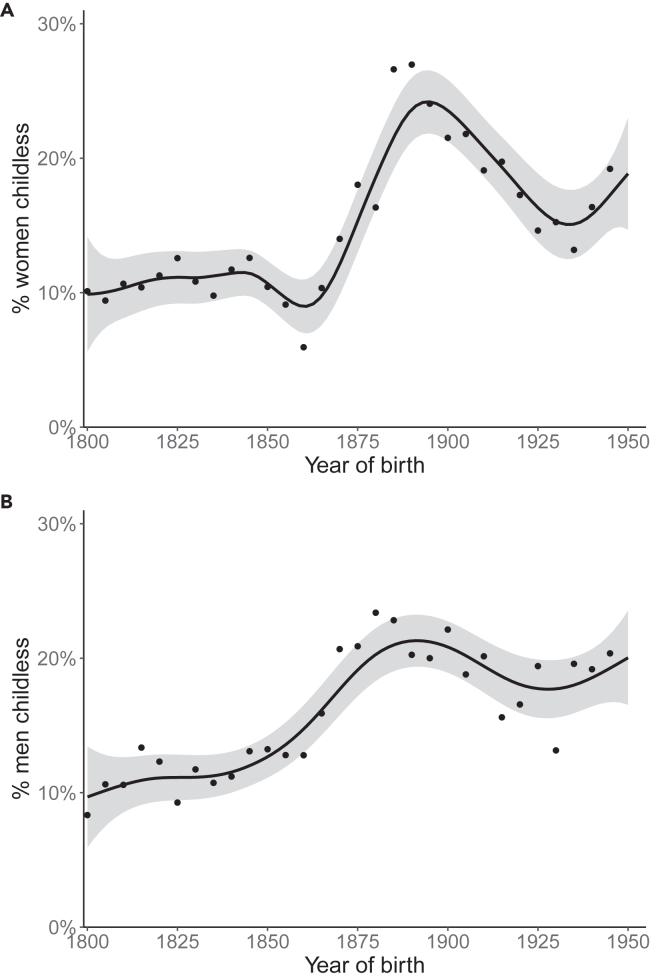
Historical trends of lifetime childlessness by sex Proportions of (A) women and (B) men lifetime childless. The x axis denotes the year of birth. The line is a smoothed conditional line with 95% confidence limits indicated by the gray area. Points indicate the mean childlessness in 5-year birth cohorts. N_A_ = 9,154 and N_B_ = 8,415.

Across the 150-year study period, the mean proportion of lifetime-childless individuals (having survived until 45 years of age and not being registered as the parent of any child) was 14.6 percent of the total sample, but there is strong temporal variation in childlessness (χ2 = 257.5, ref.df = 299, edf = 8.161, *p* < 2e-16; ref.df = reference degrees of freedom used in statistical inference). The proportion of childless individuals stayed relatively low, around 10%, until the birth cohort of 1850. Lifetime childlessness increased thereafter, peaking in the 1890 birth cohort. At the start of the 20th century, the proportions of men and women who had no children decreased until the cohorts of the 1930s, after which lifetime childlessness again steadily increased until the end of the study period (Figure 2).

Men were slightly more often childless than women: overall 15.2% of men were childless compared to 14.1% of women. Women had stronger temporal trends (χ2 = 164.50, ref.df = 299, edf = 8.375, *p* < 2e-16; Figure 2A) than men, who showed a nearly steady increase in childlessness throughout the study period, with only a weak increase in men born in the 1890s and a weak decrease until the cohorts born in the 1930s (χ2 = 104.10, ref.df = 299, edf = 5.17, *p* < 2e-16; Figure 2B). However, the difference in the shape of the trend did not significantly differ by sex (χ2 = 6.764, ref.df = 299, edf = 3.641, *p* = 0.0916).

### Historical trends of childlessness by SES

As predicted, we detected notable differences in fertility resilience during crises when comparing socioeconomic groups. Our results indicate the highest fertility resilience among landowners and those with socially highly ranked professions, but significant disturbances in fertility during crises among the lowest socioeconomic group as highlighted by the differences in trends in childlessness. High SES includes landowners, clergy, and noblemen in historical times and highly educated individuals in modern times. Moderate SES includes, for example, tenant farmers and craftsmen. Low SES consists of servants, dependent lodgers, less educated, and unemployed. Childlessness was more likely in low-SES groups than in high-SES groups: of those with high SES, 8.2 percent were lifetime childless compared to 10.2 percent childless of those with moderate SES and 19.8 percent of those with low SES. In the high-SES group, lifetime childlessness steadily and moderately increased until the end of the study period (χ2 = 28.56, ref.df = 292, edf = 3.54, *p* = 1.97e-6). In the moderate SES-group, the proportion of lifetime-childless individuals peaked among those born at the end of the 18^th^ century and only weakly decreased after that (χ2 = 82.07, ref.df = 299, edf = 4.314, *p* < 2e-16). In the low-SES group, the proportion of childless individuals was much higher compared to those with high and moderate SES and also exhibited larger temporal fluctuations (χ2 = 40.59, ref.df = 299, edf = 7.618, *p* = 4.87e-6). When comparing the trends between SES groups with the high-SES group as the reference, the trend of the moderate-SES group did not significantly differ from the trend of the high-SES group (χ2 = 0.696, ref.df = 182, edf = 1.437, *p* = 0.624), but the low-SES group strongly differed from the high-SES group by showing a considerably more non-linear trend (χ2 = 24.918, ref.df = 299, edf = 6.741, *p* = 0.000119).

**Figure 3:**
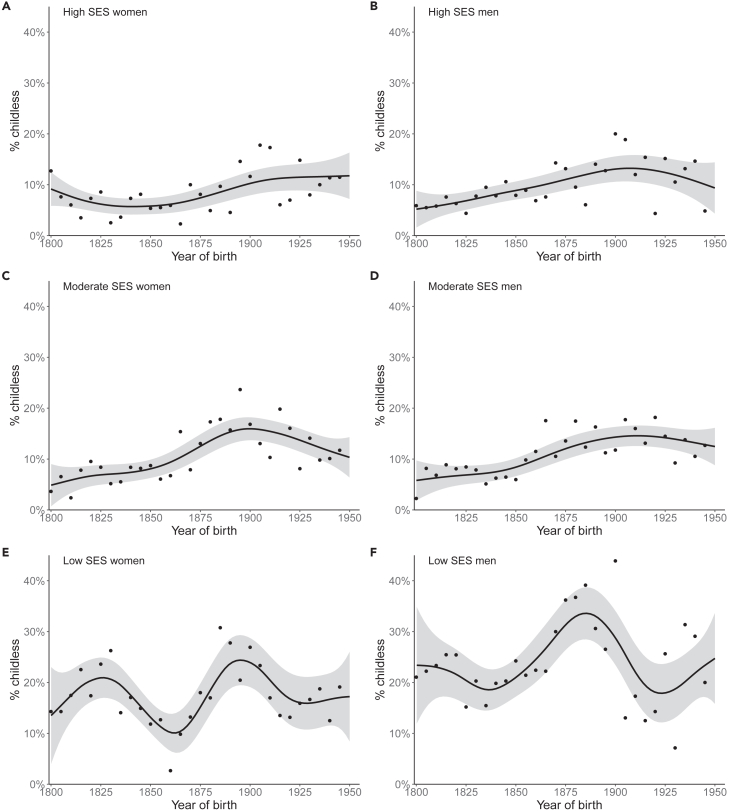
Historical trends of lifetime childlessness by sex and socioeconomic status (SES) Proportion of (A) high-SES women, (B) high-SES men, (C) moderate-SES women, (D) moderate-SES men, (E) low-SES women, and (F) low-SES men who are lifetime childless. The x axis denotes the year of birth. The line is a smoothed conditional line with 95% confidence limits indicated by the gray area. Points indicate the mean childlessness in 5-year birth cohorts. N_A_ = 2,573, N_B_ = 2,562, N_c_ = 3,436, N_D_ = 3,220, N_E_ = 2,153, and N_F_ = 1,850.

The trends among women and men were highly similar in different socioeconomic groups. Among those with high SES, 7.5% of women and 9% of men never reproduced and lifetime childlessness slightly increased toward the end of the study period (women: χ2 = 16.38, ref.df = 278, edf = 3.237, *p* = 0.000547, Figure 3A; men: χ2 = 18.31, ref.df = 256, edf = 2.834, *p* = 0.00036; Figure 3B). Among those with moderate SES, 10.1% of women and 10.3% of men did not reproduce and lifetime childlessness followed a similar trend with a nearly linear increase but a slight bump in the 1890–1900 birth cohorts (women: χ2 = 45.45, ref.df = 295, edf = 3.703, *p* < 2e-16, Figure 3C; men: χ2 = 36.00, ref.df = 286, edf = 2.994, *p* < 2e-16; Figure 3D). Low-SES men were more often childless than low-SES women: 23.2% and 16.8%, respectively. In the low-SES group, trends in both male and female childless fluctuated largely during the study period (women: χ2 = 27.66, ref.df = 299, edf = 6.91, *p* = 7.85e-5, Figure 3E; men: χ2 = 25.18, ref.df = 299, edf = 5.936, *p* = 0.000105; Figure 3F), although male childlessness peaked earlier than female, in the 1880–1890 birth cohorts.

### Socioeconomic differences in trends of marrying and childlessness in marriage

**Figure 4:**
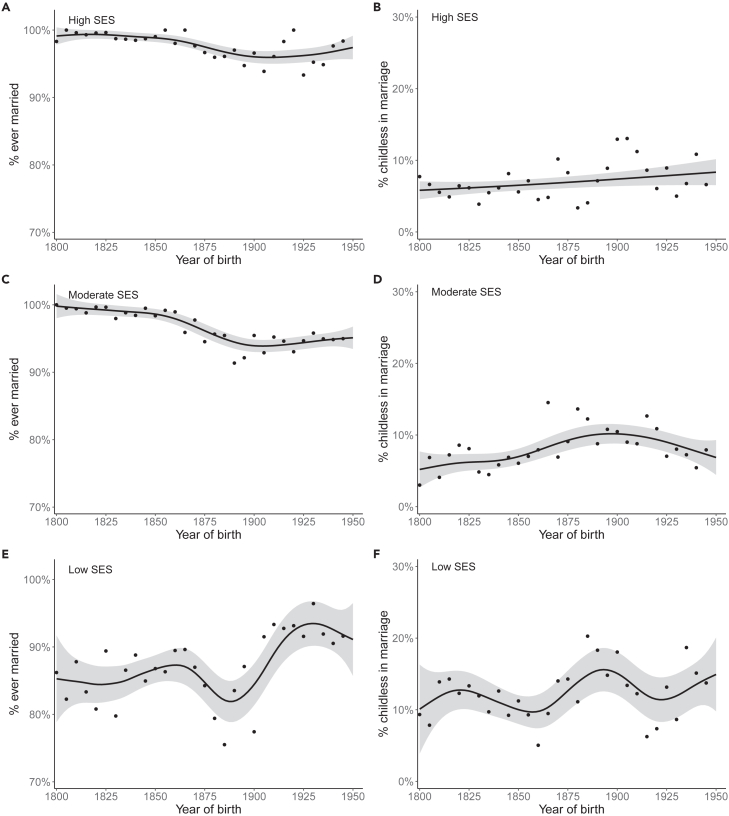
Historical trends of marriage and childlessness in wedlock by socioeconomic status (SES) Proportion of (A) high-SES ever married, (B) high-SES childless in marriage, (C) moderate-SES ever married, (D) moderate-SES childless in marriage, (E) low-SES ever married, and (F) low-SES childless in marriage. The x axis denotes the year of birth. The line is a smoothed conditional line with 95% confidence limits indicated by the gray area. Points indicate the mean proportion of ever married and childless in 5-year birth cohorts. N_A_ = 5,135, N_B_ = 5,047, N_c_ = 6,656, N_D_ = 6,457, N_E_ = 4,003, and N_F_ = 3,471.

In total, 92.1% of both women and men that survived to age 45 married at least once. Temporal trends of childlessness emerged in individuals that married at least once (χ2 = 64.05, ref.df = 299, edf = 4.494, *p* < 2e-16). The proportion of lifetime childless among married individuals was much lower than in the whole study population (Figure S3A), although the time trends of childlessness were similar, if less pronounced, in marriage (Figure S3). These trends emerged both in women (χ2 = 38.00, ref.df = 299, edf = 4.693, *p* < 2e-16; Figure S3B) and in men (χ2 = 26.75, ref.df = 277, edf = 2.644, *p* = 1.07e-06; Figure S3C), although in men it was nearly linear. Trends of marriage and childlessness in marriage varied by SES (Figure 4). Only 1.7% of the high-SES group never married, whereas 3.0% of the moderate-SES group and 13.3% of the low-SES group never married. In wedlock, lifetime childlessness was most common in the low-SES group: 6.7% of those with high SES, 7.7% of those with moderate SES, and 12.0% of individuals with low SES never reproduced. The high- and moderate-SES groups experienced a permanent decrease in marriage rates around the 1890 birth cohort, whereas the marriage rate among those with low SES increased rapidly from the 1890 birth cohorts.

### Socioeconomic differences in fertility resilience

**Figure 5:**
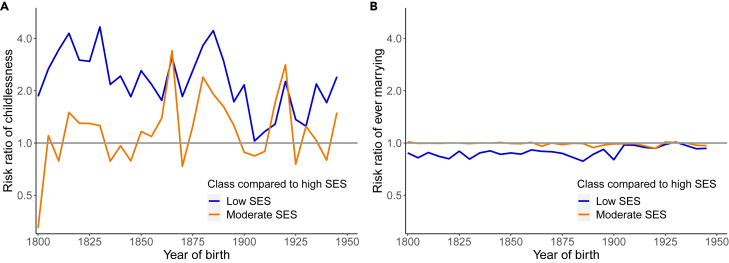
Risk ratios of childlessness and ever marrying comparing low and moderate SES to high SES Risk ratio of (A) childlessness and (B) ever marrying. The x axis denotes the year of birth. The spacing of ticks on the y axis is on a log2 scale. Colored line indicates the risk ratio in 5-year birth cohorts. The gray line is the baseline (high SES).

Fertility resilience fluctuated most over time in the low-SES group (Figures 3 and 4). Using the high-SES group as a baseline, we further explored these differences by calculating and plotting risk ratios of childlessness and ever marrying among those with low and moderate SES compared to high SES (Figure 5). The risk ratio of the low-SES group in comparison to the high-SES group for lifetime childlessness had a mean of 2.44 (SD = 0.96) but highly fluctuated over time, varying between 1.03 and 4.66. The risk ratio of the moderate-SES group was, on average, 1.29 (SD = 0.65) and fluctuated between 0.32 and 3.40. The risk ratio for ever marrying in the low-SES group in comparison to the high-SES group had a mean of 0.89 (SD = 0.058) and varied between 0.79 and 1.01. The risk ratio for ever marrying in the moderate-SES group was, on average, 0.99 (SD = 0.020) and varied between 0.93 and 1.02. The risk ratios of both the moderate- and low-SES groups compared with the high-SES group converged to near 1 in the latest birth cohorts, indicating an equal likelihood of lifetime childlessness and marrying in these cohorts.

### Additional results

#### Historical trends in possible underlying factors

Several factors appeared to underlie the observed temporal patterns of childlessness. First, the mean number of children among those who reproduced per 5-year birth cohort was highly negatively correlated with the mean proportion of childless per 5-year birth cohort (r = −0.674, df = 28, *p* = 4.503e-05; Figure S1A). Second, the mean proportion of ever married per 5-year birth cohort was highly negatively correlated with the mean proportion of lifetime-childless individuals (r = −0.934, df = 28, *p* = 4.613e-14, Figure S1B). Third, the mean age at first marriage per 5-year birth cohort was not overall associated with the mean proportion of childless individuals (r = 0.187, df = 28, *p* = 0.3214) but correlated positively in birth cohorts 1800–1930 (r = 0.436, df = 25, *p* = 0.02297, Figure S1C). Fourth, the proportion of SES groups in our sample changed during the study period, with the share of the high-SES group decreasing from 45% in the 1800 birth cohort to 23% in 1945 birth cohort. The mean proportion of lifetime childless per 5-year birth cohort was negatively correlated with the mean proportion of individuals with high SES (r = −0.568, df = 28, *p* = 0.001054) and positively correlated with the mean proportion of individuals with moderate SES (r = 0.478, df = 28, *p* = 0.007489, Figure S1D).

#### Historical trends of childlessness in different regions of Finland

In total, 15% were childless in northern and eastern Finland, 12% in central Finland, and 16.5% in southwest Finland. In northern and eastern Finland, childlessness slightly increased during the study period (χ2 = 11.16, ref.df = 297, edf = 4.672, *p* = 0.0271; Figure S2A). In central and southwestern Finland, the temporal trends followed a similar pattern, but, in southwestern Finland, childlessness increased more and the peak occurred earlier than in central Finland (central: χ2 = 92.39, ref.df = 299, edf = 6.64, *p* < 2e-16, Figure S2B; southwest: χ2 = 178.3, ref.df = 299, edf = 6.386, *p* < 2e-16; Figure S2C).

## Discussion

Major social crises can affect reproduction and resilience in childbearing behavior—what we here call fertility resilience. Fertility resilience shapes the future of individuals, families, lineages, and populations. Becoming a parent is one of the key indicators of fertility behavior overall, and the relative contribution of childlessness to fertility rates often grows as fertility declines.^7^

We investigated for the first time how fertility resilience varied within a population across a 150-year period of gradual societal change and demographic transition. The study followed trends in Finland from a time filled with socioeconomic crises, notably the Finnish famine of 1866–1868 and the Finnish Civil War and Spanish flu epidemic of 1918–1920. We focused on trends in childlessness, here defined as failure to become a parent among those who lived until 45 years of age. Using unique, individual-level data in a large sample of Finnish kin lineages including people born between 1800 and 1950, we explored how rates of lifetime childlessness varied by birth cohort, sex, and SES and examined underlying factors. Results indicate that social standing highly influences trends in childlessness, as low SES was linked to a higher likelihood of childlessness and larger fluctuations in time. Our hypothesis of lower fertility resilience among low-SES individuals was thus confirmed. Indeed, the fluctuation in childlessness over time appeared to be driven by the effects of crises on low socioeconomic class in this population, whereas a general trend of increasing lifetime childlessness was related to a steady increase of this feature in high- and moderate-SES group.

Results for general trends in lifetime childlessness showed the expected gradual increase in childlessness, from 10% to around 20%, during the 150-year study period. The temporal trends in childlessness seemed to parallel the trends observed in other Western countries.^8^^,^^9^^,^^10^ Childlessness increased considerably after the 1860s birth cohorts and peaked for both men and women born in 1890–1900. A similar peak has been reported for many Western countries,^8^^,^^9^^,^^10^ with the First World War and the Great Depression as probable causes.^8^^,^^9^ While Finland did not take part in WWI and the Great Depression was very short lived,^29^^,^^33^ the Finnish Civil War in 1918 resulted in excess deaths in men.^34^ This likely explains the childlessness peak in our sample and justifies the higher peak in lifetime childlessness among women, who faced greater difficulty in finding a spouse. In contrast, the Baby Boom after WWII demonstrates a decrease in childlessness in birth cohorts of 1920–1925. Other crises do not seem to have as drastic influences, which is may be explained by our use of cohort outcomes rather than period rates of fertility or childlessness. Crises can influence childlessness in many cohorts, from those born during the crisis to those in their late fertile years, and these influences can differ in magnitude and direction, rendering the fertility effects of these crises less pronounced on cohort than on period fertility rates. Our results in timing of fluctuations in childlessness align with other studies, showing that timing of trends in childlessness is highly similar in different countries.

We discovered that individuals with low SES were much more often childless than those with moderate or high SES, and this difference was especially pronounced in men. Low SES is known to be linked to poorer health and a lower chance to marry, compared to high and moderate SES.^9^^,^^10^^,^^21^^,^^22^ We found both lower likelihood of ever marrying and a higher likelihood of childlessness in marriage among those with low SES compared to those with higher status, indicating that both difficulties finding a spouse and poorer health may underlie the lower likelihood of ever reproducing among those with low SES. However, it is also possible that those with low resources could consciously choose not to marry or have children, although it is unlikely as living outside a marital union was not approved. Our results also indicate higher variance in the male risk of childlessness, as we expected. Overall, shape of trend did not differ between men and women whereas low SES showed significantly more temporal non-linear variation than high SES. Due to higher male mortality, sex differences would likely have been greater had we studied all adults, not only those surviving to age 45.

We hypothesized that fertility resilience would be high among individuals with many resources and low for individuals with less resources. This hypothesis was supported by our results, as we observed a modest and stable increase in childlessness in the high socioeconomic group, but strong temporal fluctuations in the low-SES group. Among those with high SES, cohort probabilities of childlessness and marriage fluctuated between the 5-year birth cohorts, yet the smoothed conditional line traced the long-lasting pattern of decreased fertility due to demographic transition, industrialization, and urbanization.^5^^,^^6^ Among those with low SES, however, both the smoothed conditional lines and risk ratios of childlessness and ever marrying by age 45 exhibited major fluctuations throughout the study period, especially in the earlier birth cohorts. Thus, effects of specific crises seem to differ and diffuse across birth cohorts with two exceptions. First, women born just before the Finnish famine had a lower likelihood of childlessness. This contrasting finding reflects the fact that crises can also promote earlier ages as childbearing, since age at first birth is often positively correlated with expected lifespan.^35^ Furthermore, those in poorest health, many of whom would have remained childless, may have not survived the famine. Second, birth cohorts that were young adults during the Finnish civil war were more likely childless than earlier cohorts.

As resilience is the ability to resist changes and recover from them,^1^ our main result indicates that a lack of social and economic resources results in low fertility resilience. Interestingly, the risk ratios stabilized near baseline for the last birth cohorts of our study period, suggesting a diminished importance of SES for fertility resilience toward the middle of the 20^th^ century. However, socioeconomic differences also decreased during our study period, due to rising living standards and the first steps toward a welfare state.^36^ It is hence also possible that the latest cohorts in our study faced fewer hardships, leading to smaller differences between socioeconomic groups. Our sample does not include cohorts born after 1950, but we know that, in later birth cohorts in Finland, the period rate of childlessness has continued to increase^37^ and cohort fertility has decreased.^38^ As childlessness has recently increased most among low educated,^25^ it is possible that SES differences in fertility resilience also have increased.

In line with previous research, trends in overall fertility, marriage, and SES coincided with the historical trends in lifetime childlessness, with the trend of marriage showing a nearly linear negative relationship with childlessness and family size being highly negatively correlated as well. The temporal trend in the proportion of individuals with high SES correlated negatively with the trend in childlessness. The proportion of high status increased in the 1860 birth cohorts, as the Finnish famine of 1866–1868 most heavily impacted those with the least resources,^17^^,^^18^ but subsequently decreased as Finnish society changed rapidly due to land reforms and industrialization.^39^ These changes in the societal class structure are reflected in the total childlessness rate in our study population. Historical trends in lifetime childlessness also coincided with the historical trends of average family size among individuals that had at least one child. This result indicates that, at least within marriage, childlessness may also be related to fertility control and cultural changes in postponing reproduction.^9^ Marriage patterns, especially trends in ever marrying, strongly correlated with trends in childlessness, as the proportion of ever married mirrored the proportion of childlessness, confirming earlier research on the relevance of marriage trends as a strong predictor for fertility trends.^40^ Age at first marriage was associated with childlessness, but only until the birth cohorts of the 1920s. Timing of marriage has been previously found to influence marital fertility.^41^ However, as trends in childlessness in wedlock are quite weak, it seems that trends in ever marrying influence childlessness more than the timing of marriage, and these trends varied most by SES.

Our study has many strengths. First, we examined the temporal trends of childlessness in the same family lineages, so trends are not due to sampling different lineages at different times. Second, we have a very long study period spanning through much of the demographic transition and several major crises which impacted birth cohorts, the sexes, and socioeconomic classes differently. Third, our data are collected from the population register and therefore provide unbiased estimations of fertility. Fourth, our dataset allowed the examination of trends in fertility in both males and females and included detailed and reliable information on SES, which is rare. The utility of our data can be seen in the similarity of trends in childlessness with previously published registry data.^8^^,^^9^

In sum, this study shows that fertility resilience in a Northern European country strongly varied by SES, with low SES linked with low resilience as indicated by strong fluctuations in childlessness during a time of repeated hardships. In addition to lifetime childlessness, these fluctuations in times of crises appeared in both the likelihood of remaining unmarried and that of remaining childless in wedlock. Temporal trends in both marriage and family size were related to trends in lifetime childlessness, stressing that both factors are important causes. Today, declining fertility and escalating ecological and geopolitical crises have heightened the relevance of demographic resilience. Trends in lifetime childlessness were in this study driven by the effects of socioeconomic crises on people with fewer resources. Even though our results suggested diminished importance of SES for fertility resilience in the birth cohorts of the mid-20^th^ century, social and economic inequality has since started to increase.^36^^,^^42^ Therefore, such differential vulnerability among socioeconomic groups is relevant for the future of the human population. Preventing inequality in family formation and reproduction represents a key challenge for population resilience to crises.

### Limitations of the study

Our study has some limitations. The data are derived from population registers that record all births, marriages, and deaths independently in different parishes. Thus, although we managed to follow many individuals from birth to death even when they migrated to another parish, some were also censored before age 45 and excluded from the data. Furthermore, it is important to keep in mind that, due to high mortality and low lifespan throughout most of the study period, the individuals having reached 45 years and studied here do not represent the entire adult population. Nevertheless, our estimates seem to be very similar to previous estimates.^8^^,^^9^^,^^11^ This criterion could also inflate the differences between SES groups, but this did not seem to be the case: when flexing the requirement of survival for those that did reproduce, the difference between SES groups persisted (6.8% childless in high SES group, 16.4% childless in low SES group). The last birth cohort included in our study was born in 1945–1949, during the onset of the Baby Boom, and entered adulthood during the late Baby Boom, which was characterized by unusually low childlessness and age at marriage. Therefore, these cohorts cannot be regarded as an endpoint for long-term trends in childlessness. In future, data collection expanding further into the end of the 20^th^ century could reveal more about these trends.

## STAR★Methods

### Key resources table

**Table:** 

REAGENT or RESOURCE	SOURCE	IDENTIFIER
**Deposited data**		

Data used in this study	Available through the lead contact	Milla Salonen milla.salonen@utu.fi

**Software and algorithms**		

Code used in this study	Supplementary material	Supplementary material: R code
R 4.2.2	R Core Team	https://cran.r-project.org/

### Resource availability

#### Lead contact

Further information and requests for resources should be directed to and will be fulfilled by the lead contact, Milla Salonen (milla.salonen@utu.fi).

#### Materials availability

This study did not generate new unique materials.

#### Data and code availability

• Pseudonymized data reported in this paper will be shared by the lead contact upon request.
• All original code is available in this paper’s supplemental information.
• Any additional information required to reanalyze the data reported in this paper is available from the lead contact upon request.

### Method details

#### Study population

We studied the historical trends in childlessness in a demographic dataset derived from population registers across Finland. Information was gathered from the parish records of the Lutheran Church, which meticulously maintained registers of all births, movements, marriages, and deaths in the country since the 17th century and maintaining this registry became mandatory by law for the church in 1749.^43^ These records are extremely high quality. Our dataset encompasses survival and reproductive details from lineages originating from four distinct geographical areas and eight parishes: Hiittinen, Kustavi and Rymättylä, Southwest Finland region; Ikaalinen and Tyrvää, Pirkanmaa region; Pulkkila, Northern Ostrobothnia region; and Jaakkima and Rautu, Karelia region. In general, the primary source of livelihood for these communities was farming, with fishing supplementing livelihoods in coastal areas, thereby enhancing food predictability along the coast.^44^ These lineages were collected prospectively, by first selecting mothers born 1600–1750 in these eight parishes and then, collecting information of their marriages and children for as many generations as was possible to follow. When members of the lineage migrated, their records in the destination parish were accessed and compiled. In total, the dataset contains over 100,000 people. However, as mortality was high, many did not survive until adulthood.

In this study, we included individuals that were born between 1800 and 1950, survived until at least 45 years of age, and whose full life history was known, meaning that likely all their children were recorded. After 1950, we lack information of SES and thus, we were unable to include these cohorts. Our sample included individuals born and reproduced both before and during the major onset of the demographic transition, allowing us to examine the trends in relation to this major, long term and still ongoing process. In total, our sample included 17569 individuals, 9154 women and 8415 men (Table S1). Men may sire biological children which are not officially registered to them; however, we estimate this proportion to be tiny, around one percent.^45^^,^^46^ Finland was a traditional, largely agrarian and Lutheran country in which extramarital births were very rare throughout the study period.^47^

We utilized the number of children in examining historical trends in fertility and for deriving whether individuals were childless or not. Lifetime childlessness was defined as having survived until at least 45 years of age and had no registered children. Only around 2.5% of men and 0.09% of women had their first child after that age in our dataset, making it extremely rare that we would wrongly classify individuals that were censored after that.

The level and trends of childlessness may relate to an individual’s socioeconomic status (SES) and therefore we categorized individuals into three SES groups based on their occupation around age 30: high SES, moderate SES, and low SES, based on their own and their first spouse’s occupation. We sorted all the possible occupations into these three classes separately in the historical (born before 1850) and modern (born after 1850) parts of the dataset, as the status related to some occupations, such as landowner changes with time. This sorting was done manually considering the likely status achieved in society. In the historical part, sorting mostly reflected landownership: high SES included landowners, but also noblemen and clergy, whereas moderate SES included tenant farmers (and craftsmen) and low SES included servants and dependent lodgers who did not have access to land. This classification has captured key differences in lifespan, marriage patterns, and childbearing.^23^^,^^48^ In the modern part, high SES included highly educated and white-collar workers, moderate SES included lower-grade non-manual laborers and skilled manual laborers and low SES included unskilled manual laborers. This classification is commonly used and reflects status differences in society, which are related to, for example, physical and mental health and mortality.^49^^,^^50^^,^^51^ For examining the trends in the proportion of SES groups, we calculated the proportion of SES groups in the 5-year birth cohorts in our dataset.

For studying the historical trends in the number of children, we only included those individuals in our sample who reproduced at least once, resulting in 14997 individuals in this second sample. For examining the age at first marriage, we only included individuals that were married at some point of their lives and whose exact date of the first marriage was known, a total of 14408 individuals. For examining the trends in SES groups, we included only individuals whose SES was known, a total of 15794 individuals. Finally, for examining the trends in different regions of the country, we included those individuals whose parish of birth was known and was in Finland (or in Karelia, a region annexed to Russia after the Second World War), a total of 17073 individuals (Table S1).

### Quantification and statistical analysis

All analyses and visualizations were conducted in R version 4.2.2.^52^ Generalized additive models (GAM) were fitted to examine the significance of historical trends in childlessness and in underlying factors with the package *mgcv*.^53^^,^^54^ We used the variable under examination (childlessness, number of children, ever married, and age at first marriage) as the outcome and exact date of birth as the explanatory variable. For smoothing basis, we used the shrinkage version of cubic regression splines (bs=”cs”) with the REML smoothing parameter estimation method and basis dimension as 300, which we tested to be adequate. For examining the differences in historical trends between subgroups (SES groups and sexes), we also fitted GAM models that included the subgroups as an ordered factor, as this allows the comparison of the smoothed trends. These models used high SES and women as the reference categories. The same options were used for smoothing basis as in other GAM models. For examining the differences in fertility resilience between SES groups, we calculated the risk ratios (relative risk) for childlessness and ever marrying for comparison of low SES with high SES and moderate SES with high SES. We plotted the trends in childlessness in the whole population and in subpopulations, for example, for men and women and for SES groups separately with *ggplot2*.^55^

Pearson correlation coefficients were used to analyze the pairwise correlations in possibly underlying trends and childlessness trends. For this analysis, we calculated the mean proportion of ever married, age at first marriage, and number of children for each 5-year birth cohort between 1800 and 1950 and correlated these with the mean proportion of childlessness for each birth year.

We also plotted the trends in possible underlying factors. We used the R package *ggplot2*^55^ to visualize these trends with the mean proportion of childless individuals, proportion of ever married, age at first marriage, and number of children plotted as a scatterplot and also included smoothed conditional lines. We used GAM as the smoothing method with the same properties as explained previously. Trends in proportions of SES groups were visualized with an area plot and risk ratios with a line plot. We used the package *scales*^56^ to scale the y axis in childlessness plots to percents, package *patchwork*^57^ to combine the plots into panels, and packages *dplyr*,^58^ *plyr*,^59^ and *reshape2*^60^ for handling the data.
