# Supplementary material for: Fertility resilience varies by socioeconomic status and sex: Historical trends in childlessness across 150 years
Source: iScience. 2024 Jun 9;27(7):110227. doi: 10.1016/j.isci.2024.110227 (PMC11253507; doi:10.1016/j.isci.2024.110227)
Supplement: Document S1. Figures S1–S3, Table S1, and Data S1 [file mmc1.pdf]

iScience, Volume *27*

Supplemental information

Fertility resilience varies by socioeconomic status and sex: Historical trends in childlessness across 150 years

Milla Salonen, Mirkka Lahdenperä, Anna Rotkirch, and Virpi Lummaa

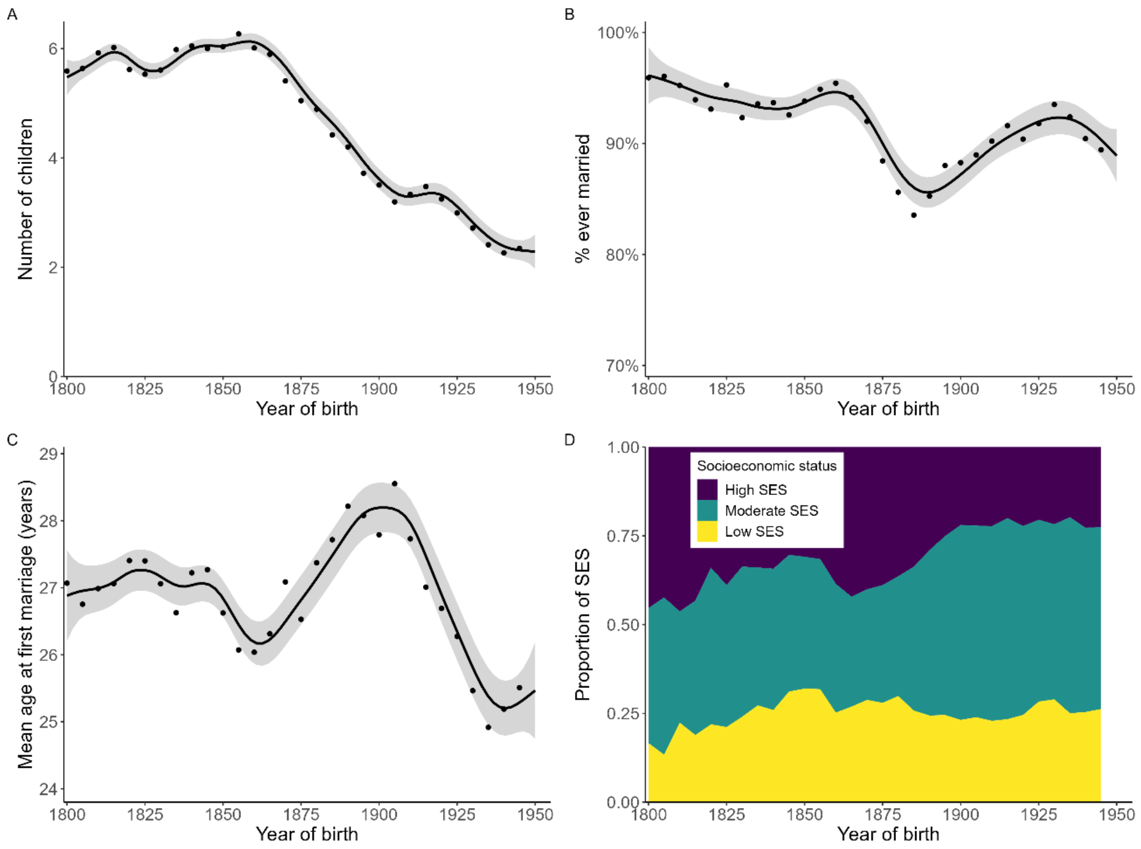

Figure S1: Historical trends in possible underlying factors, related to Results. A) number of children among those that reproduced, B) proportion ever married, C) mean age at first marriage (years), and D) proportion of SES groups (high, moderate, and low). The x-axis denotes the year of birth. The line (A, B, and C) is a smoothed conditional line with 95% confidence limits indicated by the grey area. Points (and areas in D) indicate the mean in 5-year birth cohorts. N_A = 14997, N_B = 17569, N_c = 15154, N_D = 15794.

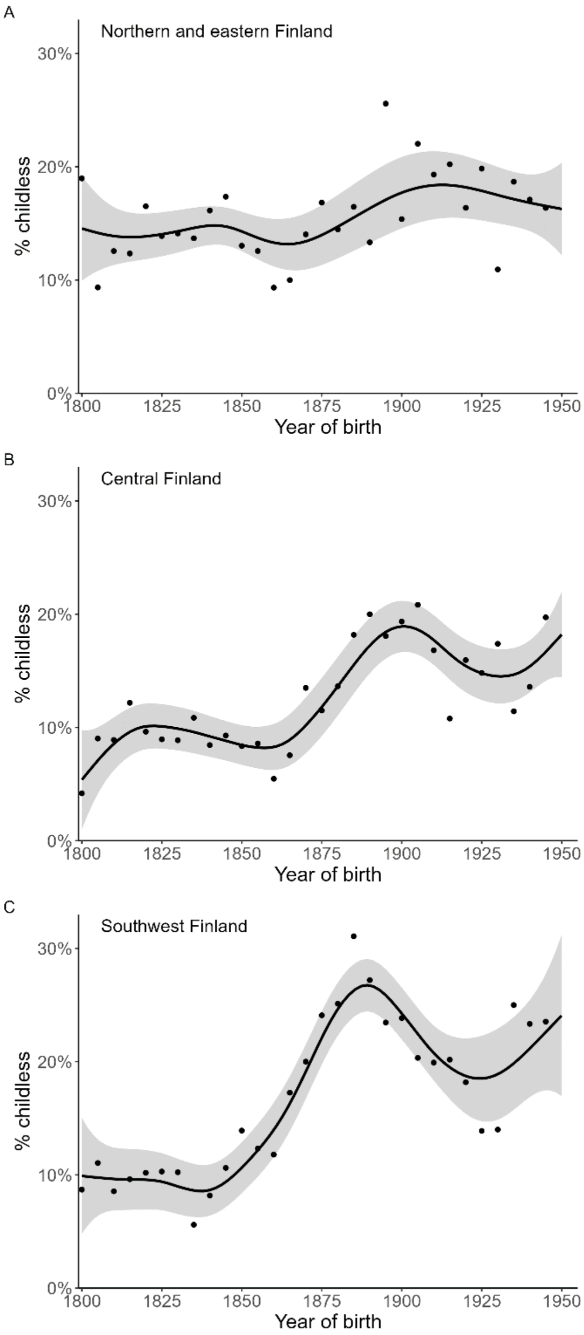

Figure S2: Historical trends of lifetime childlessness in different regions of Finland, related to Results. Proportion of childless in A) northern and eastern Finland, B) central Finland, and C) southwest Finland. The x-axis denotes the year of birth. The line is a smoothed conditional line with 95% confidence limits indicated by the grey area. Points indicate the mean childlessness in 5-year birth cohorts. N_A = 5100, N_B = 6251, N_c = 5722.

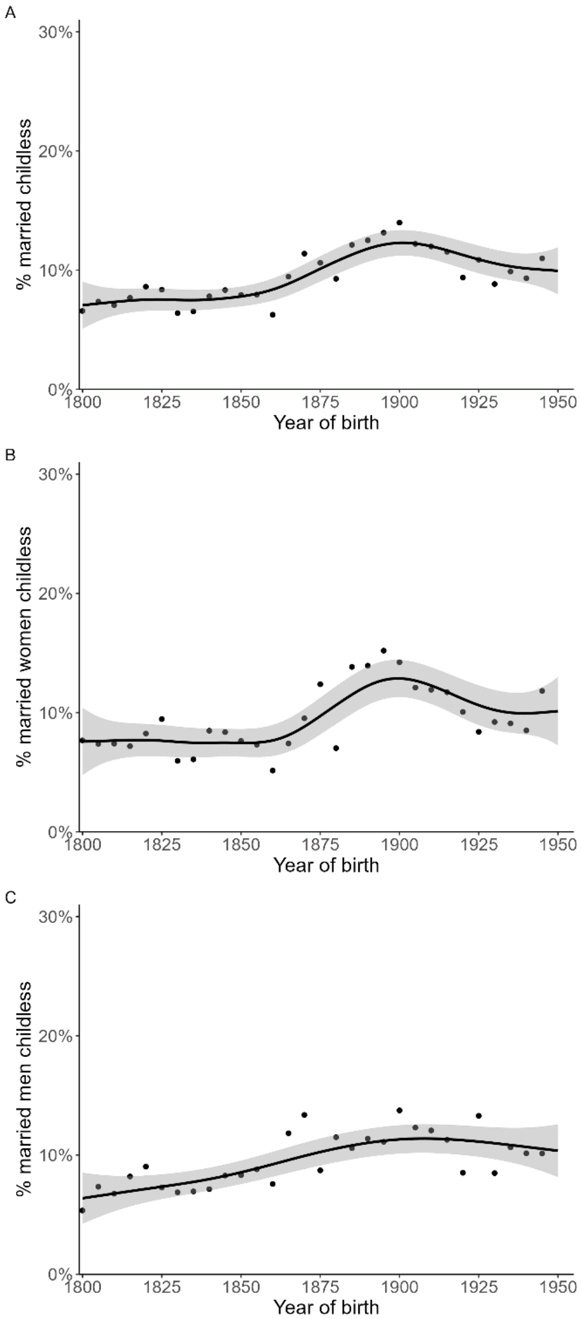

Figure S3: Historical trends of childlessness in marriage, related to Results. Proportion of A) married childless, B) married women childless, and C) married men childless. The x-axis denotes the year of birth. Line is a smoothed conditional line with 95% confidence limits indicated by the grey area. Points indicate the mean childlessness in 5-year birth cohorts. N_A = 16174, N_B = 8347, N_c = 7827.

Table S1: Descriptive statistics, related to Results.

Dataset	Cohort	N
Full dataset		17569
	Childless	2572
	Not childless	14997
	Women	9154
	Men	8415
	Ever married	16174
	Never married	1395
	1800-1804 birth cohort	539
	1805-1809 birth cohort	481
	1810-1814 birth cohort	565
	1815-1819 birth cohort	693
	1820-1824 birth cohort	724
	1825-1829 birth cohort	741
	1830-1834 birth cohort	729
	1835-1839 birth cohort	916
	1840-1844 birth cohort	1012
	1845-1849 birth cohort	1077
	1850-1854 birth cohort	1050
	1855-1859 birth cohort	703
	1860-1864 birth cohort	569
	1865-1869 birth cohort	427
	1870-1874 birth cohort	487
	1875-1879 birth cohort	458
	1880-1884 birth cohort	403
	1885-1889 birth cohort	474
	1890-1894 birth cohort	441
	1895-1899 birth cohort	467
	1900-1904 birth cohort	486
	1905-1909 birth cohort	571
	1910-1914 birth cohort	592
	1915-1919 birth cohort	369
	1920-1924 birth cohort	354
	1925-1929 birth cohort	341
	1930-1934 birth cohort	339
	1935-1939 birth cohort	460
	1940-1944 birth cohort	439
	1945-1949 birth cohort	662
Ever married		16174
	Childless	1468
	Not childless	14706
Socioeconomic status known		15794

High SES		5135
	Childless	423
	Not childless	4712
	Women	2573
	Men	2562
	Ever married	5047
	Never married	88
Moderate SES		6656
	Childless	681
	Not childless	5975
	Women	3436
	Men	3220
	Ever married	6457
	Never married	199
Low SES		4003
	Childless	791
	Not childless	3212
	Women	2153
	Men	1850
	Ever married	3471
	Never married	532
Parish of birth known and in Finland		17073
	Central Finland	6251
	Northeastern Finland	5100
	Southwest Finland	5722
Married with known age of 1st marriage		14408

Data S1: R code, related to STAR Methods.

```
#### PACKAGES ####

# for filtering data
library(dplyr)
# for basic plots
library(ggplot2)
# for scaling percentages in ggplots
library(scales)
# for combining ggplot2 plots into panels
library(patchwork)
# for GAM
library(mgcv)
# 5 year cohort
library(plyr)
# long format
library(reshape2)



#### READ FINAL DATA ####


data <- read.table()


#### Filter childlessness dataset:
# age at 1st marriage >= 15
# full life history
# lifespan at least 45
# exact birthdate between 1800 and 1950
# length of marriages more than 0 and less than 30

fulldata <- data %>%
  filter(!mar1age <= 15 | is.na(mar1age)) %>%
  filter(fullifehi == 1 & lifespanatleast >= 45) %>%
  filter(exabirth >= 1800 & exabirth < 1950) %>%
  filter(marr1length >= 0 | is.na(marr1length)) %>%
  filter(marr2length >= 0 | is.na(marr2length)) %>%
  filter(marr3length >= 0 | is.na(marr3length)) %>%
  filter(marr4length >= 0 | is.na(marr4length)) %>%
  filter(marriage.length >= 0 & marriage.length <= 30 | is.na(marriage.length))




#### PLOTS AND GAM ####

# all plots in birth cohorts (except fig 1)
```

```
#### .. Figure 1: births and deaths from Statistics Finland ####

# statistics from Statistics Finland
stats <- read.table()
stats

# line plot of birth and death rates
birth.death <- ggplot(data=stats, aes(y=death_rate,x=Year)) +
  geom_rect(aes(xmin=1875, xmax=1996, ymin=-Inf, ymax=Inf), fill = "grey",alpha=0.01) +
  geom_line(linewidth=0.7,color="red") +
  geom_line(aes(y=birth_rate,x=Year),color="black",linewidth=0.7) +
  labs(y="Crude death/birth date per 1000 people",x="Year") +
  geom_vline(xintercept = c(1808, 1833, 1866, 1918,1942), linetype="dotted", linewidth
= 0.3) +
  annotate("text", x = 1806, y = 79, label = "Finnish War",angle=90,hjust=1) +
  annotate("text", x = 1831, y = 79, label = "Epidemics and famine",angle=90,hjust=1) +
  annotate("text", x = 1864, y = 79, label = "Finnish famine",angle=90,hjust=1) +
  annotate("text", x = 1916, y = 79, label = "Finnish Civil War",angle=90,hjust=1) +
  annotate("text", x = 1940, y = 79, label = "World War II",angle=90,hjust=1) +
  annotate("text", x = 1877, y = 2, label = "Demographic transition",hjust=0) +
  theme(axis.title=element_text(size=16),axis.text=element_text(size=14,hjust=1),
        axis.line=element_line(linewidth=0.5),
        plot.background = element_rect(fill="white"),
        panel.background = element_rect(fill="white"),
        panel.grid=element_blank(),
        plot.margin=margin(15,10,10,10)) +
  coord_cartesian(ylim=c(0,81),xlim=c(1799,1996),expand=F)



# save the plot
ggsave(filename="births-deaths.pdf",birth.death,device="pdf",
       width=2000, height=1500,units="px")
ggsave(filename="births-deaths.png",birth.death,device="png",
       width=2000, height=1500,units="px")





#### .. Figure 2: childlessness in men & women ####



#### childlessness in women


# filter women
women <- fulldata %>%
  filter(sex == 2)
nrow(women)

# 14.1% of women remained childless
prop.table(table(women$childless))

# mean proportion of childless women
women.mean <- aggregate(women$childless,list(women$cohort.5),mean)
```

```r
# smoothed conditional line plot with averages of 5 year birth cohorts included as poin
ts
child.wom <- ggplot(data=women, aes(y=childless,x=exabirth)) +
  geom_smooth(method="gam",color="black",formula=y~s(x, bs="cs",k=300)) +
  geom_point(women.mean,mapping=aes(x=Group.1, y=x)) +
  labs(y="% women childless",x="Year of birth") +
  scale_y_continuous(labels=percent) + scale_x_continuous(breaks=seq(1800,1950,25)) +
  theme(axis.title=element_text(size=16),axis.text=element_text(size=14),
        axis.line=element_line(linewidth=0.5),
        plot.background = element_rect(fill="white"),
        panel.background = element_rect(fill="white"),
        panel.grid=element_blank()) +
  coord_cartesian(ylim=c(0,0.31),xlim=c(1799,1955),expand=F)


# GAM
gam.women <- gam(childless ~ s(exabirth,bs="cs",k=300),method="REML",
                 family= "binomial",data=women)
summary(gam.women)




#### childlessness in men

# filter men from data
men <- fulldata %>%
  filter(sex==1)
nrow(men)

# 15.2% of men remained childless
prop.table(table(men$childless))

# mean proportion of childless men each year
men.mean <- aggregate(men$childless,list(men$cohort.5),mean)

# smoothed conditional line plot with averages of 5 year birth cohorts included as poin
ts
child.men <- ggplot(data=men, aes(y=childless,x=exabirth)) +
  geom_smooth(method="gam",color="black",formula=y~s(x, bs="cs",k=300)) +
  geom_point(men.mean,mapping=aes(x=Group.1, y=x)) +
  labs(y="% men childless",x="Year of birth") +
  scale_y_continuous(labels=percent) + scale_x_continuous(breaks=seq(1800,1950,25)) +
  theme(axis.title=element_text(size=16),axis.text=element_text(size=14),
        axis.line=element_line(linewidth=0.5),
        plot.background = element_rect(fill="white"),
        panel.background = element_rect(fill="white"),
        panel.grid=element_blank()) +
  coord_cartesian(ylim=c(0,0.31),xlim=c(1799,1955),expand=F)

# GAM
gam.men <- gam(childless ~ s(exabirth,bs="cs",k=300),method="REML",
               family= "binomial",data=men)
summary(gam.men)
```

```r
#### plots
childless.sexes <- child.wom + child.men +
  plot_layout(ncol=1, nrow=2) + plot_annotation(tag_levels="A")
ggsave(filename="childless-men-women.png",childless.sexes,device="png",
       width=2000, height=3000,units="px")
ggsave(filename="childless-men-women.pdf",childless.sexes,device="pdf",
       width=2000, height=3000,units="px")







#### ..... difference between sexes ####

# ordered sex with women specified as the reference
fulldata$sex_o <- ordered(fulldata$sex,levels=c("2","1"))

# gam including sex and the non-linear trend of birth date by ordered sex
gam.sex <- gam(childless ~ sex + s(exabirth,bs="cs",k=300) +
                 s(exabirth,bs="cs",k=300, by=sex_o),
               method="REML", family="binomial", data=fulldata)
summary(gam.sex)








#### .. Figure 3: childlessness in social classes in women & men ####


# filter high SES women
class1.w <- women %>% filter(combined_socialclass == 1)

# 7.5% childless
prop.table(table(class1.w$childless))

# mean proportion of childless women in birth cohorts in high SES
class1.mean.w <- aggregate(class1.w$childless,list(class1.w$cohort.5),mean)

# smoothed conditional line plot with averages of 5 year birth cohorts included as poin
ts
childless.class1.w <- ggplot(data=class1.w, aes(y=childless,x=exabirth)) +
  geom_smooth(method="gam",color="black",formula=y~s(x, bs="cs",k=300)) +
  geom_point(class1.mean.w,mapping=aes(x=Group.1, y=x)) +
  labs(y="% childless",x="Year of birth") +
  annotate("text", x = 1806, y = 0.44, label = "High SES women",hjust=0, size=5) +
  scale_y_continuous(labels=percent) + scale_x_continuous(breaks=seq(1800,1950,25)) +
  theme(axis.title=element_text(size=16),axis.text=element_text(size=14),
        axis.line=element_line(linewidth=0.5),
        plot.background = element_rect(fill="white"),
        panel.background = element_rect(fill="white"),
        panel.grid=element_blank()) +
  coord_cartesian(ylim=c(0,0.45),xlim=c(1799,1955),expand=F)

# GAM
gam.class1.w <- gam(childless ~ s(exabirth,bs="cs",k=300),method="REML",
```

```
                    family="binomial", data=class1.w)
summary(gam.class1.w)




# filter moderate SES women
class2.w <- women %>% filter(combined_socialclass == 2)

# 10.1% childless
prop.table(table(class2.w$childless))

# mean proportion of childless women in birth cohorts in moderate SES
class2.mean.w <- aggregate(class2.w$childless,list(class2.w$cohort.5),mean)

# smoothed conditional line plot with averages of 5 year birth cohorts included as poin
ts
childless.class2.w <- ggplot(data=class2.w, aes(y=childless,x=exabirth)) +
  geom_smooth(method="gam",color="black",formula=y~s(x, bs="cs",k=300)) +
  geom_point(class2.mean.w,mapping=aes(x=Group.1, y=x)) +
  labs(y="% childless",x="Year of birth") +
  scale_y_continuous(labels=percent) + scale_x_continuous(breaks=seq(1800,1950,25)) +
  annotate("text", x = 1806, y = 0.44, label = "Moderate SES women",hjust=0, size=5) +
  theme(axis.title=element_text(size=16),axis.text=element_text(size=14),
        axis.line=element_line(linewidth=0.5),
        plot.background = element_rect(fill="white"),
        panel.background = element_rect(fill="white"),
        panel.grid=element_blank()) +
  coord_cartesian(ylim=c(0,0.45),xlim=c(1799,1955),expand=F)

# GAM
gam.class2.w <- gam(childless ~ s(exabirth,bs="cs",k=300),method="REML",
                    family="binomial", data=class2.w)
summary(gam.class2.w)




# filter low SES women
class3.w <- women %>% filter(combined_socialclass == 3)

# 16.8% childless
prop.table(table(class3.w$childless))

# mean proportion of childless women in birth cohorts in low SES
class3.mean.w <- aggregate(class3.w$childless,list(class3.w$cohort.5),mean)

# smoothed conditional line plot with averages of 5 year birth cohorts included as poin
ts
childless.class3.w <- ggplot(data=class3.w, aes(y=childless,x=exabirth)) +
  geom_smooth(method="gam",color="black",formula=y~s(x, bs="cs",k=300)) +
  geom_point(class3.mean.w,mapping=aes(x=Group.1, y=x)) +
  labs(y="% childless",x="Year of birth") +
  scale_y_continuous(labels=percent) + scale_x_continuous(breaks=seq(1800,1950,25)) +
  annotate("text", x = 1806, y = 0.44, label = "Low SES women",hjust=0, size=5) +
  theme(axis.title=element_text(size=16),axis.text=element_text(size=14),
        axis.line=element_line(linewidth=0.5),
        plot.background = element_rect(fill="white"),
```

```
        panel.background = element_rect(fill="white"),
        panel.grid=element_blank()) +
  coord_cartesian(ylim=c(0,0.45),xlim=c(1799,1955),expand=F)

# GAM
gam.class3.w <- gam(childless ~ s(exabirth,bs="cs",k=300),method="REML",
                    family="binomial",data=class3.w)
summary(gam.class3.w)




#### men

# filter high SES men
class1.m <- men %>% filter(combined_socialclass == 1)

# 9% childless
prop.table(table(class1.m$childless))

# mean proportion of childless men in birth cohorts in high SES
class1.mean.m <- aggregate(class1.m$childless,list(class1.m$cohort.5),mean)

# smoothed conditional line plot with averages of 5 year birth cohorts included as poin
ts
childless.class1.m <- ggplot(data=class1.m, aes(y=childless,x=exabirth)) +
  geom_smooth(method="gam",color="black",formula=y~s(x, bs="cs",k=300)) +
  geom_point(class1.mean.m,mapping=aes(x=Group.1, y=x)) +
  labs(y="% childless",x="Year when 25 years of age") +
  scale_y_continuous(labels=percent) + scale_x_continuous(breaks=seq(1800,1950,25)) +
  annotate("text", x = 1806, y = 0.44, label = "High SES men",hjust=0, size=5) +
  theme(axis.title=element_text(size=16),axis.text=element_text(size=14),
        axis.line=element_line(linewidth=0.5),
        plot.background = element_rect(fill="white"),
        panel.background = element_rect(fill="white"),
        panel.grid=element_blank()) +
  coord_cartesian(ylim=c(0,0.45),xlim=c(1799,1955),expand=F)

# GAM
gam.class1.m <- gam(childless ~ s(exabirth,bs="cs",k=300),method="REML",
                    family="binomial", data=class1.m)
summary(gam.class1.m)




# filter moderate SES men
class2.m <- men %>% filter(combined_socialclass == 2)

# 10.3% childless
prop.table(table(class2.m$childless))

# mean proportion of childless men in birth cohorts in moderate SES
class2.mean.m <- aggregate(class2.m$childless,list(class2.m$cohort.5),mean)

# smoothed conditional line plot with averages of 5 year birth cohorts included as poin
ts
```

```
childless.class2.m <- ggplot(data=class2.m, aes(y=childless,x=exabirth)) +
  geom_smooth(method="gam",color="black",formula=y~s(x, bs="cs",k=300)) +
  geom_point(class2.mean.m,mapping=aes(x=Group.1, y=x)) +
  labs(y="% childless",x="Year of birth") +
  scale_y_continuous(labels=percent) + scale_x_continuous(breaks=seq(1825,1975,25)) +
  annotate("text", x = 1806, y = 0.44, label = "Moderate SES men",hjust=0, size=5) +
  theme(axis.title=element_text(size=16),axis.text=element_text(size=14),
        axis.line=element_line(linewidth=0.5),
        plot.background = element_rect(fill="white"),
        panel.background = element_rect(fill="white"),
        panel.grid=element_blank()) +
  coord_cartesian(ylim=c(0,0.45),xlim=c(1799,1955),expand=F)

# GAM
gam.class2.m <- gam(childless ~ s(exabirth,bs="cs",k=300),method="REML",
                    family="binomial", data=class2.m)
summary(gam.class2.m)



# filter low SES men
class3.m <- men %>% filter(combined_socialclass == 3)

# 23.2% childless
prop.table(table(class3.m$childless))

# mean proportion of childless men in birth cohorts in low SES
class3.mean.m <- aggregate(class3.m$childless,list(class3.m$cohort.5),mean)

# smoothed conditional line plot with averages of 5 year birth cohorts included as poin
ts
childless.class3.m <- ggplot(data=class3.m, aes(y=childless,x=exabirth)) +
  geom_smooth(method="gam",color="black",formula=y~s(x, bs="cs",k=300)) +
  geom_point(class3.mean.m,mapping=aes(x=Group.1, y=x)) +
  labs(y="% childless",x="Year of birth") +
  annotate("text", x = 1806, y = 0.44, label = "Low SES men",hjust=0, size=5) +
  scale_y_continuous(labels=percent) + scale_x_continuous(breaks=seq(1800,1950,25)) +
  theme(axis.title=element_text(size=16),axis.text=element_text(size=14),
        axis.line=element_line(linewidth=0.5),
        plot.background = element_rect(fill="white"),
        panel.background = element_rect(fill="white"),
        panel.grid=element_blank()) +
  coord_cartesian(ylim=c(0,0.45),xlim=c(1799,1955),expand=F)

gam.class3.m <- gam(childless ~ s(exabirth,bs="cs",k=300),method="REML",
                    family="binomial", data=class3.m)
summary(gam.class3.m)



## plots

childless.soclass.sexes <- childless.class1.w + childless.class1.m +
  childless.class2.w + childless.class2.m +
  childless.class3.w +  childless.class3.m +
  plot_layout(ncol=2, nrow=3) + plot_annotation(tag_levels="A")
ggsave(filename="childless-socialclass-sexes.png",childless.soclass.sexes,device="png",
       width=4000, height=4500,units="px")
ggsave(filename="childless-socialclass-sexes.pdf",childless.soclass.sexes,device="pdf",
       width=4000, height=4500,units="px")
```

```r
#### ..... difference between classes ####

# ordered social class with high SES specified as the reference
fulldata$combined_socialclass_o <- ordered(fulldata$combined_socialclass,
                                           levels=c("1","2","3"))


# gam including SES and the non-linear trend of birth date by ordered SES
gam.ses <- gam(childless ~ combined_socialclass + s(exabirth,bs="cs",k=300) +
                 s(exabirth,bs="cs",k=300, by=combined_socialclass_o),
               method="REML", family="binomial", data=fulldata)
summary(gam.ses)




#### .. Figure 4: marriage and childlessness in marriage in soclasses ####

# filter high SES
class1 <- fulldata %>% filter(combined_socialclass == 1)

# 1.7% never married
prop.table(table(class1$evermarried))

# mean proportion of married in birth cohorts in high SES
marriage.class1.mean <- aggregate(class1$evermarried,list(class1$cohort.5),mean)

# smoothed conditional line plot with averages of 5 year birth cohorts included as poin
ts
marriage.class1 <- ggplot(data=class1, aes(y=evermarried,x=exabirth)) +
  geom_smooth(method="gam",color="black",formula=y~s(x, bs="cs",k=300)) +
  geom_point(marriage.class1.mean,mapping=aes(x=Group.1, y=x)) +
  labs(y="% ever married",x="Year of birth") +
  annotate("text", x = 1806, y = 1.015, label = "High SES",hjust=0, size=5) +
  scale_y_continuous(labels=percent) + scale_x_continuous(breaks=seq(1800,1950,25)) +
  theme(axis.title=element_text(size=16),
        axis.text=element_text(size=14),
        axis.line=element_line(linewidth=0.5),
        plot.background = element_rect(fill="white"),
        panel.background = element_rect(fill="white"),
        panel.grid=element_blank()) +
  coord_cartesian(ylim=c(0.69,1.03),xlim=c(1799,1955),expand=F)




## childlessness in wedlock in high SES

# filter those that were married
class1.marr <- class1 %>%
  filter(evermarried == 1)
```

```
# 6.7% childless in ever married
prop.table(table(class1.marr$childless))

# mean proportion of married childless in birth cohorts in high SES
marr.child.class1.mean <- aggregate(class1.marr$childless,list(class1.marr$cohort.5),me
an)

# smoothed conditional line plot with averages of 5 year birth cohorts included as poin
ts
childless.m.class1 <- ggplot(data=class1.marr, aes(y=childless,x=exabirth)) +
  geom_smooth(method="gam",color="black",formula=y~s(x, bs="cs",k=3)) +
  geom_point(marr.child.class1.mean,mapping=aes(x=Group.1, y=x)) +
  labs(y="% childless in marriage",x="Year of birth") +
  annotate("text", x = 1806, y = 0.3, label = "High SES",hjust=0, size=5) +
  scale_y_continuous(labels=percent) + scale_x_continuous(breaks=seq(1800,1950,25)) +
  theme(axis.title=element_text(size=16),
        axis.text=element_text(size=14),
        axis.line=element_line(linewidth=0.5),
        plot.background = element_rect(fill="white"),
        panel.background = element_rect(fill="white"),
        panel.grid=element_blank()) +
  coord_cartesian(ylim=c(-0.01,0.31),xlim=c(1799,1955),expand=F)



# filter moderate SES
class2 <- fulldata %>% filter(combined_socialclass == 2)

# 3.0% never married
prop.table(table(class2$evermarried))

# mean proportion of married in birth cohorts in moderate SES
marriage.class2.mean <- aggregate(class2$evermarried,list(class2$cohort.5),mean)

# smoothed conditional line plot with averages of 5 year birth cohorts included as poin
ts
marriage.class2 <- ggplot(data=class2, aes(y=evermarried,x=exabirth)) +
  geom_smooth(method="gam",color="black",formula=y~s(x, bs="cs",k=300)) +
  geom_point(marriage.class2.mean,mapping=aes(x=Group.1, y=x)) +
  labs(y="% ever married",x="Year of birth") +
  annotate("text", x = 1806, y = 1.015, label = "Moderate SES",hjust=0, size=5) +
  scale_y_continuous(labels=percent) + scale_x_continuous(breaks=seq(1800,1950,25)) +
  theme(axis.title=element_text(size=16),
        axis.text=element_text(size=14),
        axis.line=element_line(linewidth=0.5),
        plot.background = element_rect(fill="white"),
        panel.background = element_rect(fill="white"),
        panel.grid=element_blank()) +
  coord_cartesian(ylim=c(0.69,1.03),xlim=c(1799,1955),expand=F)



## childlessness in wedlock in moderate SES

# filter those that were married
class2.marr <- class2 %>%
  filter(evermarried == 1)

# 6.7% childless in ever married
prop.table(table(class2.marr$childless))
```

```
# mean proportion of married in birth cohorts in high SES
marr.child.class2.mean <- aggregate(class2.marr$childless,list(class2.marr$cohort.5),me
an)

# smoothed conditional line plot with averages of 5 year birth cohorts included as poin
ts
childless.m.class2 <- ggplot(data=class2.marr, aes(y=childless,x=exabirth)) +
  geom_smooth(method="gam",color="black",formula=y~s(x, bs="cs",k=300)) +
  geom_point(marr.child.class2.mean,mapping=aes(x=Group.1, y=x)) +
  labs(y="% childless in marriage",x="Year of birth") +
  annotate("text", x = 1806, y = 0.3, label = "Moderate SES",hjust=0, size=5) +
  scale_y_continuous(labels=percent) + scale_x_continuous(breaks=seq(1800,1950,25)) +
  theme(axis.title=element_text(size=16),
        axis.text=element_text(size=14),
        axis.line=element_line(linewidth=0.5),
        plot.background = element_rect(fill="white"),
        panel.background = element_rect(fill="white"),
        panel.grid=element_blank()) +
  coord_cartesian(ylim=c(-0.01,0.31),xlim=c(1799,1955),expand=F)



# filter low SES
class3 <- fulldata %>% filter(combined_socialclass == 3)

# 13.3% never married
prop.table(table(class3$evermarried))

# mean proportion of married in birth cohorts in low SES
marriage.class3.mean <- aggregate(class3$evermarried,list(class3$cohort.5),mean)

# smoothed conditional line plot with averages of 5 year birth cohorts included as poin
ts
marriage.class3 <- ggplot(data=class3, aes(y=evermarried,x=exabirth)) +
  geom_smooth(method="gam",color="black",formula=y~s(x, bs="cs",k=300)) +
  geom_point(marriage.class3.mean,mapping=aes(x=Group.1, y=x)) +
  labs(y="% ever married",x="Year of birth") +
  annotate("text", x = 1806, y = 1.015, label = "Low SES",hjust=0, size=5) +
  scale_y_continuous(labels=percent) + scale_x_continuous(breaks=seq(1800,1950,25)) +
  theme(axis.title=element_text(size=16),
        axis.text=element_text(size=14),
        axis.line=element_line(linewidth=0.5),
        plot.background = element_rect(fill="white"),
        panel.background = element_rect(fill="white"),
        panel.grid=element_blank()) +
  coord_cartesian(ylim=c(0.69,1.03),xlim=c(1799,1955),expand=F)



## childlessness in wedlock in low SES

# filter those that married
class3.marr <- class3 %>%
  filter(evermarried == 1)

# 6.7% childless in ever married
prop.table(table(class3.marr$childless))
```

```r
# mean proportion of married in birth cohorts in high SES
marr.child.class3.mean <- aggregate(class3.marr$childless,list(class3.marr$cohort.5),me
an)

# smoothed conditional line plot with averages of 5 year birth cohorts included as poin
ts
childless.m.class3 <- ggplot(data=class3.marr, aes(y=childless,x=exabirth)) +
  geom_smooth(method="gam",color="black",formula=y~s(x, bs="cs",k=300)) +
  geom_point(marr.child.class3.mean,mapping=aes(x=Group.1, y=x)) +
  labs(y="% childless in marriage",x="Year of birth") +
  annotate("text", x = 1806, y = 0.3, label = "Low SES",hjust=0, size=5) +
  scale_y_continuous(labels=percent) + scale_x_continuous(breaks=seq(1800,1950,25)) +
  theme(axis.title=element_text(size=16),
        axis.text=element_text(size=14),
        axis.line=element_line(linewidth=0.5),
        plot.background = element_rect(fill="white"),
        panel.background = element_rect(fill="white"),
        panel.grid=element_blank()) +
  coord_cartesian(ylim=c(-0.01,0.31),xlim=c(1799,1955),expand=F)



# plots
childless.soclass.marriage <- marriage.class1 + childless.m.class1 +
  marriage.class2 + childless.m.class2 + marriage.class3 + childless.m.class3 +
  plot_layout(ncol=2, nrow=3) + plot_annotation(tag_levels="A")
ggsave(filename="childless-soclass-marriage.png",childless.soclass.marriage,device="png
",
       width=4000, height=4500,units="px")
ggsave(filename="childless-soclass-marriage.pdf",childless.soclass.marriage,device="pdf
",
       width=4000, height=4500,units="px")





#### .. Figure 5: risk ratios ####


# high SES used as a baseline

# 5 year cohorts

# mean proportion of childless in birth cohorts for each SES group
class1.mean <- aggregate(class1$childless,list(class1$cohort.5),mean)
class2.mean <- aggregate(class2$childless,list(class2$cohort.5),mean)
class3.mean <- aggregate(class3$childless,list(class3$cohort.5),mean)

# combine cohort averages
classes <- class1.mean %>%
  full_join(.,class2.mean,by="Group.1") %>%
  full_join(.,class3.mean, by="Group.1") %>%
  rename(class1 = x.x,
         class2 = x.y,
         class3 = x)
```

```r
# calculate risk ratios
classes <- classes %>%
  mutate("Low SES" = class3/class1,
         "Moderate SES" = class2/class1) %>%
  dplyr::select(Group.1,"Low SES", "Moderate SES")


# change to long format so that ggplot2 legends can be used
classes.long <- reshape2::melt(classes,id.var="Group.1")

# ratio plot
ratio.plot <- ggplot(data=classes.long, aes(y=value,x=Group.1, color=variable)) +
  geom_line(linewidth=1) +
  labs(y="Risk ratio of childlessness",x="Year of birth",
       color = "Class compared to high SES") +
  scale_x_continuous(breaks=seq(1800,1950,25)) +
  theme(axis.title=element_text(size=16),axis.text=element_text(size=14),
        axis.line=element_line(linewidth=0.5),
        plot.background = element_rect(fill="white"),
        panel.background = element_rect(fill="white"),
        panel.grid=element_blank(),
        legend.position= c(0.5, 0.95),
        legend.text = element_text(size=14),
        legend.title = element_text(size=14)) +
  geom_hline(yintercept = 1, linetype="solid", linewidth = 1,color="grey60") +
  coord_cartesian(ylim=c(0,6),xlim=c(1799,1955),expand=F) +
  scale_color_manual(values=c("blue3", "darkorange2"))




#### risk ratio for marriage

# mean proportion of married in birth cohorts for each SES group
class1.mean.marr <- aggregate(class1$evermarried,list(class1$cohort.5),mean)
class2.mean.marr <- aggregate(class2$evermarried,list(class2$cohort.5),mean)
class3.mean.marr <- aggregate(class3$evermarried,list(class3$cohort.5),mean)

# combine cohort averages
classes.marr <- class1.mean.marr %>%
  full_join(.,class2.mean.marr,by="Group.1") %>%
  full_join(.,class3.mean.marr, by="Group.1") %>%
  rename(class1 = x.x,
         class2 = x.y,
         class3 = x)


# calculate risk ratios
classes.marr <- classes.marr %>%
  mutate("Low SES" = class3/class1,
         "Moderate SES" = class2/class1) %>%
  dplyr::select(Group.1,"Low SES", "Moderate SES")
sd(classes.marr$`Moderate SES`)
summary(classes.marr)

# change to long format so that ggplot2 legends can be used
classes.marr.long <- reshape2::melt(classes.marr,id.var="Group.1")

# ratio plot
ratio.plot.marriage <- ggplot(data=classes.marr.long,
```

```
                                    aes(y=value,x=Group.1, color=variable)) +
  geom_line(linewidth=1) +
  labs(y="Risk ratio of ever marrying",x="Year of birth",
       color = "Class compared to high SES") +
  scale_x_continuous(breaks=seq(1800,1950,25)) +
  theme(axis.title=element_text(size=16),axis.text=element_text(size=14),
        axis.line=element_line(linewidth=0.5),
        plot.background = element_rect(fill="white"),
        panel.background = element_rect(fill="white"),
        panel.grid=element_blank(),
        legend.position= c(0.5, 0.95),
        legend.text = element_text(size=14),
        legend.title = element_text(size=14)) +
  geom_hline(yintercept = 1, linetype="solid", linewidth = 1,color="grey60") +
  coord_cartesian(ylim=c(0.7,1.1),xlim=c(1799,1955),expand=F) +
  scale_color_manual(values=c("blue3", "darkorange2"))



# plots

risk.ratios <- ratio.plot.childless + ratio.plot.marriage +
  plot_layout(ncol=2, nrow=1) + plot_annotation(tag_levels="A")
ggsave(filename="risk-ratios-marriage-chidlessness-soclass.png",risk.ratios,device="png
",
       width=4000, height=1500,units="px")
ggsave(filename="risk-ratios-marriage-chidlessness-soclass.pdf",risk.ratios,device="pdf
",
       width=4000, height=1500,units="px")





#### .. Fig. S1. Underlying variables ####


#### .... a: fertility ####

# filter out individuals with no children
children <- fulldata %>%
  filter(childless == 0)
nrow(children)

# mean number of children for 5 year birth cohorts
children.mean <- aggregate(children$kids,list(children$cohort.5),mean)

# smoothed conditional line plot with averages of 5 year birth cohorts included as poin
ts
fertility <- ggplot(data=children, aes(y=kids,x=exabirth)) +
  geom_smooth(method="gam",color="black",formula=y~s(x, bs="cs",k=300)) +
  geom_point(children.mean,mapping=aes(x=Group.1, y=x)) +
  labs(y="Number of children",x="Year of birth") +
  scale_x_continuous(breaks=seq(1800,1950,25)) +
  theme(axis.title=element_text(size=16),axis.text=element_text(size=14),
        axis.line=element_line(linewidth=0.5),
        plot.background = element_rect(fill="white"),
        panel.background = element_rect(fill="white"),
```

```
        panel.grid=element_blank()) +
  coord_cartesian(ylim=c(0,6.5),xlim=c(1799,1955),expand=F)


## GAM
gam.kids <- gam(kids ~ s(exabirth,bs="cs",k=300),method="REML", family="gaussian", data
=children)
summary(gam.kids)


# find the maximum
values.fer <- predict(gam.kids)
values.fer <- as.data.frame(values.fer)
values.fer <- cbind(children,values.fer)
summary(values.fer)

values.fer <- aggregate(values.fer$values.fer, by=list(values.fer$byear),mean)
values.fer[which.max(values.fer$x),]


### correlation in 5 year birth cohorts
fertility.childlessness <- cor.test(children.mean$x,childless.mean$x)






#### .... b: ever married ####

# mean proportion of evermarried for 5 y cohorts
marr.mean <- aggregate(fulldata$evermarried,list(fulldata$cohort.5),mean)

# smoothed conditional line plot with averages of 5 year birth cohorts included as poin
ts
married <- ggplot(data=fulldata, aes(y=evermarried,x=exabirth)) +
  geom_smooth(method="gam",color="black",formula=y~s(x, bs="cs",k=300)) +
  geom_point(marr.mean,mapping=aes(x=Group.1, y=x)) +
  labs(y="% ever married",x="Year of birth") +
  scale_y_continuous(labels=percent) + scale_x_continuous(breaks=seq(1800,1950,25)) +
  theme(axis.title=element_text(size=16),axis.text=element_text(size=14),
        axis.line=element_line(linewidth=0.5),
        plot.background = element_rect(fill="white"),
        panel.background = element_rect(fill="white"),
        panel.grid=element_blank()) +
  coord_cartesian(ylim=c(0.69,1.01),xlim=c(1799,1955),expand=F)


## GAM
gam.married <- gam(evermarried ~ s(exabirth,bs="cs",k=300),method="REML", family="binom
ial", data=fulldata)
summary(gam.married)

# find the maximum and minimum

values.marr <- predict(gam.married,type="response")
values.marr <- as.data.frame(values.marr)
values.marr <- cbind(fulldata,values.marr)
summary(values.marr)
```

```
values.marr <- aggregate(values.marr$values.marr, by=list(values.marr$cohort.5),mean)
values.marr[which.max(values.marr$x),]
values.marr[which.min(values.marr$x),]



### correlation
evermarried.childlessness <- cor.test(marr.mean$x,childless.mean$x)









#### .... c: age at first marriage ####

# filter those that were married and have age at first marriage known
marr.age.data <- fulldata %>%
  filter(evermarried == 1 & !is.na(mar1age))

# mean age of first marriage for 5 y cohorts
marr.age.mean <- aggregate(marr.age.data$mar1age,list(marr.age.data$cohort.5),mean)

# smoothed conditional line plot with averages of 5 year birth cohorts included as poin
ts
marriage.age <- ggplot(data=marr.age.data, aes(y=mar1age,x=exabirth)) +
  geom_smooth(method="gam",color="black",formula=y~s(x, bs="cs",k=300)) +
  geom_point(marr.age.mean,mapping=aes(x=Group.1, y=x)) +
  labs(y="Mean age at first marriage (years)",x="Year of birth") +
  scale_x_continuous(breaks=seq(1800,1950,25)) +
  theme(axis.title=element_text(size=16),axis.text=element_text(size=14),
        axis.line=element_line(linewidth=0.5),
        plot.background = element_rect(fill="white"),
        panel.background = element_rect(fill="white"),
        panel.grid=element_blank()) +
  coord_cartesian(ylim=c(23.8,29.1),xlim=c(1799,1955),expand=F)


## GAM
gam.marr.age <- gam(mar1age ~ s(exabirth,bs="cs",k=300),method="REML", family="gaussian
", data=marr.age.data)
summary(gam.marr.age)

# find the minimum and maximum
values.mar3 <- predict(gam.marr.age,type="response")
values.mar3 <- as.data.frame(values.mar3)
values.mar3 <- cbind(marr.age.data,values.mar3)

values.mar3 <- aggregate(values.mar3$values.mar3, by=list(values.mar3$cohort.5),mean)
values.mar3[which.max(values.mar3$x),]
values.mar3[which.min(values.mar3$x),]


### correlation

# whole data
```

```
marr.age.childlessness <- cor.test(marr.age.mean$x,childless.mean$x)

# before 1930 cohort
marr.age.childlessness.1930 <- cor.test(marr.age.mean$x[1:27],childless.mean$x[1:27])




#### .... d: trends in social classes ####


# named and ordered social class
fulldata <- fulldata %>%
  mutate(soclass_fac = case_when(combined_socialclass == 1 ~ "1-high",
                                 combined_socialclass == 2 ~ "2-moderate",
                                 combined_socialclass == 3 ~ "3-low")) %>%
  mutate(soclass_fac = factor(soclass_fac,ordered = T))

# filter those with known SES
# make dummy variables in which individuals have values 1/0
# based on whether they belong to that class or not
socdata <- fulldata %>%
  filter(!is.na(soclass_fac)) %>%
  mutate(high = ifelse(soclass_fac == "1-high",1,0),
         moderate = ifelse(soclass_fac == "2-moderate",1,0),
         low = ifelse(soclass_fac == "3-low",1,0))

# calculate the proportion of SES groups in each 5-y birth cohort
classes <- socdata  %>%
  group_by(cohort.5, soclass_fac) %>%
  summarise(n = n()) %>%
  mutate(percentage = n / sum(n))

# area plot
soc.trend <- ggplot(data = classes,aes(x = cohort.5, y = percentage, fill = soclass_fac
)) +
  geom_area(position = "fill", colour = "black", linewidth = 0, alpha = 1) +
  scale_fill_viridis_d(labels=c("High SES","Moderate SES","Low SES")) +
  labs(y="Proportion of SES", x="Year of birth",
       fill="Socioeconomic status") +
  scale_x_continuous(breaks=seq(1800,1950,25)) +
  theme(axis.title=element_text(size=16),axis.text=element_text(size=14),
        axis.line=element_line(linewidth=0.5),
        plot.background = element_rect(fill="white"),
        panel.background = element_rect(fill="white"),
        panel.grid=element_blank(),
        legend.position=c(0.25,0.85),
        legend.text = element_text(size=12),
        legend.title = element_text(size=12)) +
  coord_cartesian(ylim=c(0,1),xlim=c(1799,1955),expand=F)




# mean proportion of high SES in 5 year cohorts
childless.mean.soc <- aggregate(socdata$childless,list(socdata$cohort.5),mean)
```

```
# correlation between proportion of high SES and childless
cor.test(high.mean$x,childless.mean.soc$x)



# mean proportion of moderate SES in 5 year cohorts
moderate.mean <- aggregate(socdata$moderate,list(socdata$cohort.5),mean)

# correlation between proportion of moderate SES and childless
cor.test(moderate.mean$x,childless.mean$x)



# mean proportion of low SES in 5 year cohorts
low.mean <- aggregate(socdata$low,list(socdata$cohort.5),mean)

# correlation between proportion of low SES and childless
cor.test(low.mean$x,childless.mean$x)





# plots

underlying <- fertility + married +  marriage.age + soc.trend +
  plot_layout(ncol=2, nrow=2) + plot_annotation(tag_levels="A")
ggsave(filename="underlying-factors.pdf",underlying,device="pdf",
       width=4000, height=3000,units="px")
ggsave(filename="underlying-factors.png",underlying,device="png",
       width=4000, height=3000,units="px")







#### .. Fig S2 : childlessness in regions ####


# three regions around the original study parishes


# filter those born in Central Finland
central <- fulldata %>%
  filter(four.region == "Central")

# mean proportion of childless in 5 year cohorts
child.mean.central <- aggregate(central$childless,list(central$cohort.5),mean)

# smoothed conditional line plot with averages of 5 year birth cohorts included as poin
ts
child.central <- ggplot(data=central, aes(y=childless,x=exabirth)) +
  geom_smooth(method="gam",color="black",formula=y~s(x, bs="cs",k=300)) +
  geom_point(child.mean.central,mapping=aes(x=Group.1, y=x)) +
  labs(y="% childless",x="Year of birth") +
  annotate("text", x = 1806, y = 0.32, label = "Central Finland",hjust=0, size=5) +
```

```
  scale_y_continuous(labels=percent) + scale_x_continuous(breaks=seq(1800,1950,25)) +
  theme(axis.title=element_text(size=16),axis.text=element_text(size=14),
        axis.line=element_line(linewidth=0.5),
        plot.background = element_rect(fill="white"),
        panel.background = element_rect(fill="white"),
        panel.grid=element_blank()) +
  coord_cartesian(ylim=c(0,0.33),xlim=c(1799,1955),expand=F)

# GAM
gam.central <- gam(childless ~ s(exabirth,bs="cs",k=300),method="REML",
                   family="binomial", data=central)
summary(gam.central)



# filter those born in Southwest Finland
southwest <- fulldata %>%
  filter(four.region == "Southwest")

# mean proportion of childless in 5 year cohorts
child.mean.southwest <- aggregate(southwest$childless,list(southwest$cohort.5),mean)

# smoothed conditional line plot with averages of 5 year birth cohorts included as poin
ts
child.southwest <- ggplot(data=southwest, aes(y=childless,x=exabirth)) +
  geom_smooth(method="gam",color="black",formula=y~s(x, bs="cs",k=300)) +
  geom_point(child.mean.southwest,mapping=aes(x=Group.1, y=x)) +
  labs(y="% childless",x="Year of birth") +
  annotate("text", x = 1806, y = 0.32, label = "Southwest Finland",hjust=0, size=5) +
  scale_y_continuous(labels=percent) + scale_x_continuous(breaks=seq(1800,1950,25)) +
  theme(axis.title=element_text(size=16),axis.text=element_text(size=14),
        axis.line=element_line(linewidth=0.5),
        plot.background = element_rect(fill="white"),
        panel.background = element_rect(fill="white"),
        panel.grid=element_blank()) +
  coord_cartesian(ylim=c(0,0.33),xlim=c(1799,1955),expand=F)

# GAM
gam.southwest <- gam(childless ~ s(exabirth,bs="cs",k=300),method="REML",
                     family="binomial", data=southwest)
summary(gam.southwest)



# filter those born in Northern or Eastern Finland
northeast <- fulldata %>%
  filter(four.region == "Northern" | four.region == "Eastern")

# mean proportion of childless in 5 year cohorts
child.mean.northeast <- aggregate(northeast$childless,list(northeast$cohort.5),mean)

# smoothed conditional line plot with averages of 5 year birth cohorts included as poin
ts
child.northeast <- ggplot(data=northeast, aes(y=childless,x=exabirth)) +
  geom_smooth(method="gam",color="black",formula=y~s(x, bs="cs",k=300)) +
  geom_point(child.mean.northeast,mapping=aes(x=Group.1, y=x)) +
  labs(y="% childless",x="Year of birth") +
  scale_y_continuous(labels=percent) + scale_x_continuous(breaks=seq(1800,1950,25)) +
  annotate("text", x = 1806, y = 0.32, label = "Northern and eastern Finland",hjust=0,
```

```
size=5) +
  theme(axis.title=element_text(size=16),axis.text=element_text(size=14),
        axis.line=element_line(linewidth=0.5),
        plot.background = element_rect(fill="white"),
        panel.background = element_rect(fill="white"),
        panel.grid=element_blank()) +
  coord_cartesian(ylim=c(0,0.33),xlim=c(1799,1955),expand=F)

# GAM
gam.northeast <- gam(childless ~ s(exabirth,bs="cs",k=300),method="REML",
                     family="binomial", data=northeast)
summary(gam.northeast)




# plots

childless.regions.final <- child.northeast + child.central + child.southwest +
  plot_layout(ncol=1, nrow=3) + plot_annotation(tag_levels="A")
ggsave(filename="childless-3regions.pdf",childless.regions.final,device="pdf",
       width=2000, height=4500,units="px")
ggsave(filename="childless-3regions.png",childless.regions.final,device="png",
       width=2000, height=4500,units="px")




#### .. Fig S3: childlessness in marriage ####



### total


# 84.8% ever married
prop.table(table(fulldata$evermarried))
### of married, 9.1% remained childless
prop.table(table(filter(fulldata,evermarried==1)$childless))

# filter married
married.childless <- fulldata %>% filter(evermarried == 1)

# mean proportion of married childless each year
marr.child.mean <- aggregate(married.childless$childless,list(married.childless$cohort.
5),mean)

# smoothed conditional line plot with averages of 5 year birth cohorts included as poin
ts
child.married <- ggplot(data=married.childless, aes(y=childless,x=exabirth)) +
  geom_point(marr.child.mean,mapping=aes(x=Group.1, y=x)) +
```

```
  geom_smooth(method="gam",color="black",formula=y~s(x, bs="cs",k=300)) +
  labs(y="% married childless",x="Year of birth") +
  scale_y_continuous(labels=percent) + scale_x_continuous(breaks=seq(1800,1950,25)) +
  theme(axis.title=element_text(size=16),axis.text=element_text(size=14),
        axis.line=element_line(linewidth=0.5),
        plot.background = element_rect(fill="white"),
        panel.background = element_rect(fill="white"),
        panel.grid=element_blank()) +
  coord_cartesian(ylim=c(0,0.31),xlim=c(1799,1955),expand=F)


## GAM
gam.married.childless <- gam(childless ~ s(exabirth,bs="cs",k=300),method="REML",
                             family="binomial", data=married.childless)
summary(gam.married.childless)




### women

# 91.2% of women ever married
prop.table(table(women$evermarried))
### of married 9.0% remained childless
prop.table(table(filter(women,evermarried==1)$childless))
### of never married 66.7% remained childless
prop.table(table(filter(women,evermarried==0)$childless))


# filter married women
married.childless.wom <- women %>% filter(evermarried == 1)

# mean proportion of married childless women for 5-y birth cohorts
marr.child.mean.wom <- aggregate(married.childless.wom$childless,
                                 list(married.childless.wom$cohort.5),mean)

# smoothed conditional line plot with averages of 5 year birth cohorts included as poin
ts
child.married.wom <- ggplot(data=married.childless.wom, aes(y=childless,x=exabirth)) +
  geom_point(marr.child.mean.wom,mapping=aes(x=Group.1, y=x)) +
  geom_smooth(method="gam",color="black",formula=y~s(x, bs="cs",k=300)) +
  labs(y="% married women childless",x="Year of birth") +
  scale_y_continuous(labels=percent) + scale_x_continuous(breaks=seq(1800,1950,25)) +
  theme(axis.title=element_text(size=16),axis.text=element_text(size=14),
        axis.line=element_line(linewidth=0.5),
        plot.background = element_rect(fill="white"),
        panel.background = element_rect(fill="white"),
        panel.grid=element_blank()) +
  coord_cartesian(ylim=c(0,0.31),xlim=c(1799,1955),expand=F)

## GAM
gam.married.childless.wom <- gam(childless ~ s(exabirth,bs="cs",k=300),method="REML",
                                 family="binomial", data=married.childless.wom)
summary(gam.married.childless.wom)
```

```
### men

# 93.0% of men ever married
prop.table(table(men$evermarried))
### of married 9.2% remained childless
prop.table(table(filter(men,evermarried==1)$childless))
### of never married 96.3% remained childless
prop.table(table(filter(men,evermarried==0)$childless))


# filter married men
married.childless.men <- men %>% filter(evermarried == 1)

# mean proportion of married childless men each year
marr.child.mean.men <- aggregate(married.childless.men$childless,
                                 list(married.childless.men$cohort.5),mean)

# smoothed conditional line plot with averages of 5 year birth cohorts included as poin
ts
child.married.men <- ggplot(data=married.childless.men, aes(y=childless,x=exabirth)) +
  geom_point(marr.child.mean.men,mapping=aes(x=Group.1, y=x)) +
  geom_smooth(method="gam",color="black",formula=y~s(x, bs="cs",k=300)) +
  labs(y="% married men childless",x="Year of birth") +
  scale_y_continuous(labels=percent) + scale_x_continuous(breaks=seq(1800,1950,25)) +
  theme(axis.title=element_text(size=16),axis.text=element_text(size=14),
        axis.line=element_line(linewidth=0.5),
        plot.background = element_rect(fill="white"),
        panel.background = element_rect(fill="white"),
        panel.grid=element_blank()) +
  coord_cartesian(ylim=c(0,0.31),xlim=c(1799,1955),expand=F)


## GAM
gam.married.childless.men <- gam(childless ~ s(exabirth,bs="cs",k=300),method="REML",
                                 family="binomial", data=married.childless.men)
summary(gam.married.childless.men)




#### plots

childless.in.marriage <- child.married + child.married.wom + child.married.men +
  plot_layout(ncol=1, nrow=3) + plot_annotation(tag_levels="A")
ggsave(filename="childless-in-marriage.png",childless.in.marriage,device="png",
       width=2000, height=4500,units="px")
ggsave(filename="childless-in-marriage.pdf",childless.in.marriage,device="pdf",
       width=2000, height=4500,units="px")
```
